# Alternative Mating Type Configurations (a/α versus a/a or α/α) of *Candida albicans* Result in Alternative Biofilms Regulated by Different Pathways

**DOI:** 10.1371/journal.pbio.1001117

**Published:** 2011-08-02

**Authors:** Song Yi, Nidhi Sahni, Karla J. Daniels, Kevin L. Lu, Thyagarajan Srikantha, Guanghua Huang, Adam M. Garnaas, David R. Soll

**Affiliations:** Department of Biology, The University of Iowa, Iowa City, Iowa, United States of America; Duke University Medical Center, United States of America

## Abstract

Similar multicellular structures can evolve within the same organism that may have different evolutionary histories, be controlled by different regulatory pathways, and play similar but nonidentical roles. In the human fungal pathogen *Candida albicans*, a quite extraordinary example of this has occurred. Depending upon the configuration of the mating type locus (**a**/α versus **a/a** or α/α), *C. albicans* forms alternative biofilms that appear similar morphologically, but exhibit dramatically different characteristics and are regulated by distinctly different signal transduction pathways. Biofilms formed by **a**/α cells are impermeable to molecules in the size range of 300 Da to 140 kDa, are poorly penetrated by human polymorphonuclear leukocytes (PMNs), and are resistant to antifungals. In contrast, **a/a** or α/α biofilms are permeable to molecules in this size range, are readily penetrated by PMNs, and are susceptible to antifungals. By mutational analyses, **a**/α biofilms are demonstrated to be regulated by the Ras1/cAMP pathway that includes Ras1→Cdc35→cAMP(Pde2—**|**)→Tpk2(Tpk1)→Efg1→Tec1→Bcr1, and **a/a** biofilms by the MAP kinase pathway that includes Mfα→Ste2→ (Ste4, Ste18, Cag1)→Ste11→Hst7→Cek2(Cek1)→Tec1. These observations suggest the hypothesis that while the upstream portion of the newly evolved pathway regulating **a/a** and α/α cell biofilms was derived intact from the upstream portion of the conserved pheromone-regulated pathway for mating, the downstream portion was derived through modification of the downstream portion of the conserved pathway for **a**/α biofilm formation. *C. albicans* therefore forms two alternative biofilms depending upon mating configuration.

## Author Summary

Single-celled microbes can form biofilms, or aggregates of cells that adhere to one another on a surface, in response to many environmental factors. Like many microbial pathogens, the yeast *Candida albicans* can form biofilms that normally provide protective environments against antifungals, antibodies, and white blood cells, thus ensuring higher rates of survival in response to assault by drugs or the human immune system. We report that while a majority (around 90%) of *C. albicans* strains form traditional biofilms that are impermeable to molecules of low and high molecular weight, and that are impenetrable to white blood cells, a minority (around 10%) form biofilms that are both permeable and penetrable. Formation of the minority-type alternative biofilms is dictated by a change at a single genetic locus, the mating type locus. Homozygous **a**/**a** or α/α cells are mating-competent, whereas the heterozygous **a**/α cells are mating-incompetent. Cells of the mating-incompetent **a**/α genotype form the impermeable, traditional biofilm, whereas the mating-competent **a/a** or α/α genotype forms the permeable biofilm. The characteristics of **a/a** and α/α biofilms are consistent with a suggested role in mating by facilitating the transfer of hormone signals through the permeable biofilm. The two types of biofilm are also regulated by different signal transduction pathways: the **a**/α form by the Ras1/cAMP pathway, and the **a/a** or α/α forms by the MAP kinase pathway. Components of the latter pathway suggest that its downstream portion evolved from the **a**/α pathway. *C. albicans*, therefore, forms two superficially similar biofilms, exhibiting very different permeability characteristics, regulated by different signal transduction pathways, dictated by different mating type locus configurations, and serving quite different purposes in its life history.

## Introduction

During convergent evolution, similar adaptive forces can lead to similar multicellular structures through different evolutionary histories [1]. Similar multicellular structures, however, may also evolve within the same organism that appear morphologically similar, but may have different evolutionary histories and similar but not necessarily identical functions [2],[3]. Here we describe an extraordinary example of the latter, which not only reveals how an organism may use similar structures for different purposes, but also provides clues to how new signal transduction pathways evolve. We have found that depending upon the configuration of the mating type locus (**a**/α versus **a/a** or α/α), the opportunistic fungal pathogen *Candida albicans* forms alternative biofilms that are morphologically similar, but which are regulated by distinctly different signal transduction pathways and exhibit quite different characteristics consistent with different roles in the life history of this human pathogen.

The majority of *C. albicans* strains are heterozygous (**a**/α) at the mating type locus [4]–[7]. These strains cannot undergo white to opaque switching, a necessary phenotypic transition in the acquisition of mating competency, because switching as well as mating are repressed by the **a**1-α2 corepressor complex [4],[8],[9]. We can assume that because most strains of *C. albicans* in nature are **a**/α, the majority of biofilms that form in mammalian hosts, as well as on catheters and prosthetics, are **a**/α. These **a**/α biofilms appear to play the traditional role of providing a controlled environment that protects populations from outside challenges, such as host antibodies, host white blood cells, and antifungal drugs [10]–[14]. When *C. albicans* undergoes homozygosis from **a**/α to either **a/a** or α/α, it is able to switch from white to opaque, the latter the mating competent phenotype [8],[9]. Like **a**/α cells, unisexual population of white **a/a** or α/α cells form robust biofilms on the proper substratum [15],[16]. The thickness of these biofilms can be enhanced 30% to 50% by adding minority opaque cells of opposite mating types, which are a source of pheromone [15]. In the absence of opaque cells of the opposite mating type, unisexual populations of **a/a** cells self-induce biofilm formation by releasing α-pheromone and unisexual populations of α/α cells self-induce by releasing **a**-pheromone, in a mating type-nonspecific, paracrine system [16]–[18]. It has been shown in vitro that biofilms made up of white *MTL*-homozygous cells facilitate mating between embedded minority opaque **a/a** and α/α cells, which can be as far apart as 30 µm, suggesting that at least one role of *MTL*-homozygous white cell biofilms may be to protect pheromone gradients that direct chemotropism in the fusion process [15],[19].

By mutational analysis, we recently identified the major components of the pathway that regulate pheromone-induced biofilm formation by white cells, from the pheromone signal through the transduction pathway and the targeted transcription factor Tec1 [15]–[24]. We found that α-pheromone first induces white **a/a** cells to adhere to a plastic or silicone elastomer surface to form a basal yeast cell layer, the first step in biofilm formation [25], and then induces biofilm maturation, which includes the formation of hyphae oriented vertically to the substratum and the deposition of an extracellular polymolecular matrix [15]–[24]. The upper portion of the white cell pheromone response pathway includes the same pheromone signals, pheromone receptors, trimeric G-protein complex, MAP kinase cascade, and MAP kinase scaffold as the upper portion of the opaque cell pheromone response pathway in the mating process [17],[18],[20]–[23]. This common upper portion of the pathway in white cells, however, then targets the transcription factor Tec1 [22], rather than Cph1, the transcription factor targeted by the opaque pheromone response pathway for the mating response [26],[27]. Tec1 was demonstrated to bind to the *cis*-acting white-specific pheromone response element WPRE in the promoters of biofilm-related genes, resulting in the formation of a white cell biofilm [18],[22]. Given that **a**/α cells repress pheromone synthesis, we assumed that **a**/α biofilms, which are morphologically similar to *MTL*-homozygous biofilms, were regulated by a pathway other than the MAP kinase pathway, which regulates *MTL*-homozygous biofilm formation. Rather than the mating pheromone, it has been proposed that the signal for **a**/α biofilm formation may simply be mechanical, through contact with the proper tissue or prosthetic surface [28]. The pathway regulating **a**/α biofilm formation, however, had not been elucidated, as had the pathway for *MTL*-homozygous biofilm formation. Genes implicated by mutational analysis in the regulation of **a**/α biofilm formation included *EFG1* [29],[30], *BCR1* [28],[31], and *TEC1* [28],[31].

Here, we have explored two hypotheses related to the differences between *MTL*-heterozygous (**a**/α) and *MTL*-homozygous (**a/a,** α/α) biofilms, first that they play different roles in the life history of *C. albicans* and second that formation of each is regulated by a different signal transduction pathway. In regard to the first hypothesis, we demonstrate that **a**/α biofilms are highly impermeable to molecules in the 300 Da to 140 kDa range, are poorly penetrated by human polymorphonuclear leukocytes (PMNs), and are resistant to fluconazole treatment. Biofilms formed by **a/a** or α/α cells, on the other hand, are highly permeable to molecules in the 300 Da to 140 kDa range, are readily penetrated by human PMNs, and are highly susceptible to fluconazole. In regard to the second hypothesis, we demonstrate that the formation of biofilms by **a**/α cells is regulated by the Ras1/cAMP pathway, not the MAP kinase pathway, whereas the formation of biofilms by white **a/a** cells is regulated by the MAP kinase pathway [15]–[23], not the Ras1/cAMP pathway. Furthermore we show that during **a**/α biofilm formation, the Ras1/cAMP pathway activates the transcription factor cascade Efg1→Tec1→Bcr1. We have, therefore, demonstrated that although **a**/α and **a/a** biofilms are morphologically similar, they exhibit different phenotypic properties and are regulated by different signal transduction pathways. Our results further suggest that *MTL*-heterozygous biofilms provide the traditional protective, impermeable environment for commensalism and infection, whereas unisexual *MTL*-homozygous biofilms provide a more permeable environment that may facilitate mating [15],[19].

## Results

### **a**/α and *MTL*-homozygous (**a/a** and α/α) Biofilms Differ in Permeability, Human Leukocyte Penetrance, and Susceptibility to Fluconazole

**Figure 1:**
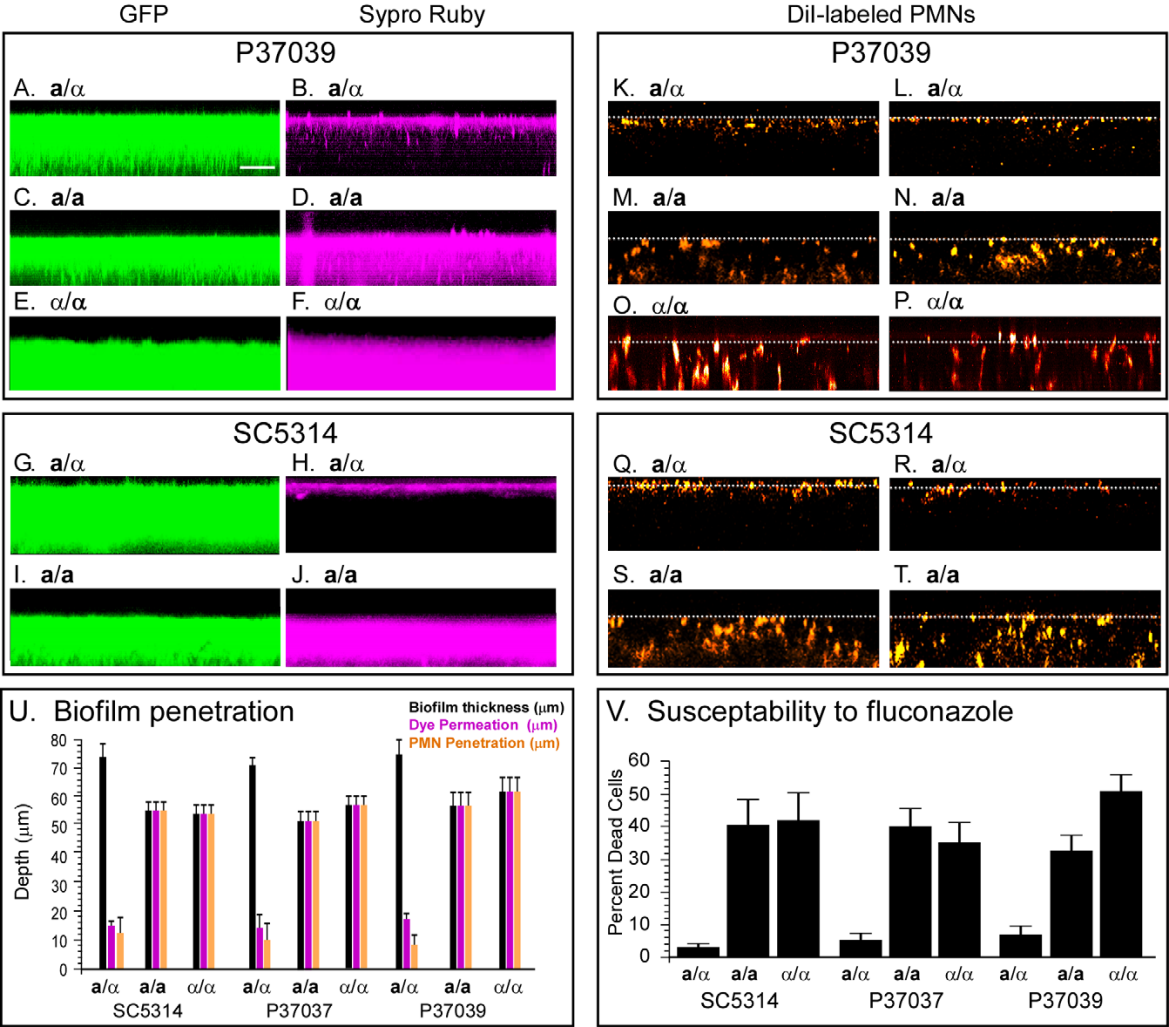
a/α biofilms differ from a/a and α/α biofilms in permeability, antifungal susceptibility, and human leukocyte penetration. Laser scanning confocal microscopy of biofilms was used to image the permeability of SYPRO Ruby and penetration by DiI-labeled human PMNs. The strains had been transformed so that each expressed green fluorescent protein (GFP) under the regulation of the actin promoter. See Table S1 for genotypes. (A, C, E, G, I) GFP fluorescence for assessing biofilm thickness and continuity. (B, D, F, H, J) SYPRO Ruby penetration 30 min after application to top of biofilms. (K through T) Penetrance by DiI-stained PMNs of two representative biofilms of each strain 3 h after the PMNs were dispersed on the top of biofilms. All images are representative of 9 biofilms for each strain and condition. (U) Biofilm thickness, permeation by SYRPO Ruby and penetration by PMNs. (V) Susceptibility to fluconazole, measured by percent cell death assessed with the dye Dead Red. In panels U and V, values are given as means ± standard derivatives. Scale bar for all biofilms (A through T) in panel A equals 50 µm.

To test for differences in permeability, we developed biofilms of **a**/α, **a/a** and α/α cells of two natural, genetically unrelated strains, P37039 and P37037, and the common laboratory strain SC5314. Biofilms were developed for 48 h on a silicone elastomer surface, then overlaid with a solution of the dye SYPRO Ruby, which has a molecular weight of 1.6 kDa [32],[33], and incubated for an additional 30 min. Biofilms were then examined without fixation by laser scanning confocal microscopy (LSCM). All nine strains examined had been transformed with a construct containing GFP driven by the constitutive actin promoter (Table S1) in order to assess formation of an intact biofilm and biofilm thickness. Side views of GFP-labeled, LSCM projections revealed that **a**/α, **a/a**, and α/α cells of strain P37039 (Figure 1A, C, and E, respectively), **a**/α, **a/a** and α/α cells of strain P37037 (unpublished data), and **a**/α and **a/a** cells of strain SC5314 (Figure 1G and I, respectively) all formed dense, relatively contiguous biofilms. The mean (± standard deviation) thickness of **a**/α biofilms of the three respective strains were 75±5 µm (*N* = 9), 71±3 µm (*N* = 9), and 74±5 µm (*N* = 9), that of white **a/a** biofilms 56±4 µm (*N* = 9), 57±3 µm (*N* = 9) and 51±3 µm (*N* = 9), and that of white α/α biofilms 61±4 µm (*N* = 9), 56±3 µm (*N* = 9), and 54±3 (*N* = 9) (Figure 1U). *MTL*-heterozygous biofilms were therefore on average approximately 28% thicker than *MTL*-homozygous biofilms. Both *MTL*-heterozygous and *MTL*-homozygous biofilms of all three strains were composed of a thin basal layer (10% to 20% of thickness) of yeast cells at the substratum and a thick upper region (80% to 90% of thickness) composed primarily of vertically oriented hyphae and extracellular matrix (unpublished data). SYPRO Ruby penetrated into only the upper 15±3% (*N* = 9) of **a**/α biofilms (Figure 1B,H,U), but penetrated through 100% (*N* = 9) of **a/a** biofilms and 100% (*N* = 9) of α/α biofilms (Figure 1D,F,J,U). Similar results were obtained for all three strains with the dye ConA (unpublished data), which has a molecular weight of 104 to 112 kDa, approximately 70 times that of SYPRO Ruby.

If **a/a** and α/α biofilms are more permeable than **a**/α biofilms, they should also be more susceptible to antifungals. To test this prediction, biofilms of the **a**/α, **a/a**, and α/α derivatives of the three strains, all expressing GFP regulated by the constitutive actin promoter (see Table S1), were developed for 48 h, then treated with 24 µg per ml of fluconazole, which has a molecular weight of 306 daltons, for 24 and 48 h. The fluconazole solution was dispersed on the top of each biofilm. Since most vital stains of metabolically active cells (i.e., living cells) could have permeability constraints in live **a**/α biofilms, we assessed viability by disrupting biofilms, then staining cells for cell death with the dye Dead Red [34]. The proportion of dead cells was 3%, 5%, and 6% in the **a**/α parental strains SC5314, P37037, and P37039, but varied between 31% and 49% in the **a/a** and α/α derivatives of these strains (Figure 1V). The average fold difference for **a**/α versus **a/a** or α/α cells was, therefore, 9-fold. These results demonstrate that **a**/α biofilms are far less susceptible to fluconazole than **a/a** or α/α biofilms.

To test for differences in human white blood cell penetrance, fresh human polymorphonuclear leukocytes (PMNs) were distributed along the surface of 48-h biofilms of the *MTL*-heterozygous strains of P37039, P37037, and SC5314, as well as their *MTL*-homozygous derivatives (Table S1), and incubated for 3 h. Prior to distribution, PMNs were stained with the vital membrane dye DiI [35],[36]. Since the excitation wavelength for imaging DiI overlaps that for GFP, transmitted light images rather than GFP images were used to assess biofilm thickness. Thickness is indicated by dotted white lines in Figure 1K–T. PMNs penetrated into only the upper 11±3% (*N* = 9) of P37039 **a**/α biofilms (Figure 1K,L,U), but into 100% of P37039 **a/a biofilms** (Figure 1M,N) and into100% of P37039 α/α biofilms (Figure 1O,P,U). Similar differences were observed between **a**/α versus **a/a** or α/α biofilms of strain P37037 (Figure 1U) and Sc5314 (Figure 1Q through T,U). These results demonstrate that in addition to dramatic differences in permeability, **a**/α biofilms are far less penetrable by human PMNs than unisexual *MTL*-homozygous (**a/a** or α/α) biofilms.

### **a**/α Biofilms Are Not Regulated by the MAP Kinase Pathway

**Figure 2:**
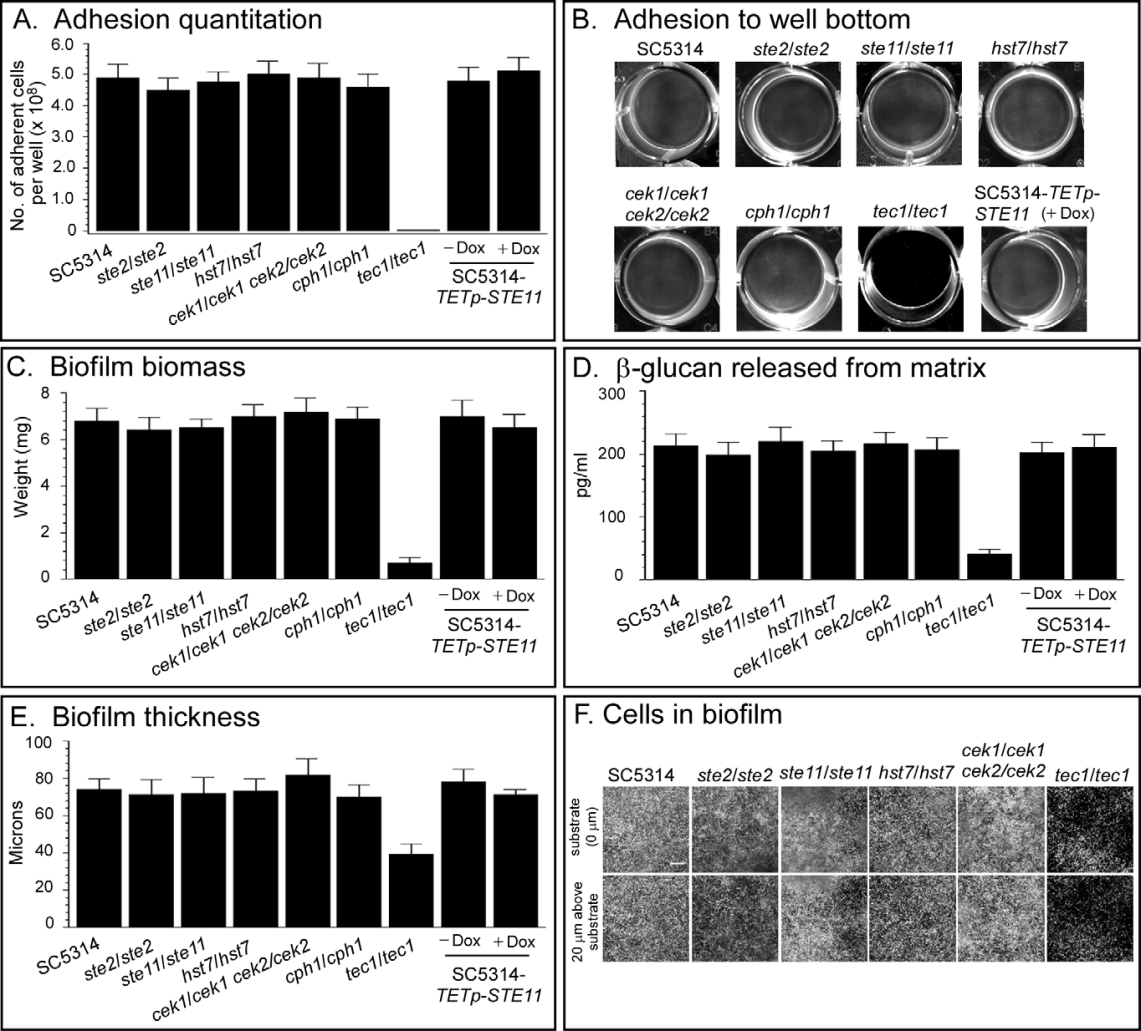
The MAP kinase response pathway plays no role in a/α biofilm formation. Deletion mutants generated in **a**/α cells of strain SC5314 for the α-pheromone receptor (*ste2/ste2*) and the MAP kinases (*ste11/ste11*, *hst7/hst7*, *cek1/cek1 cek2/cek2*), components of the upstream portion of the pheromone response pathway (see Table S2 for mutant origins and genotypes), formed normal **a**/α biofilms. The deletion mutant of *CPH1*, which encodes the targeted transcription factor in the opaque cell pheromone response, also formed normal biofilms. Overexpression of *STE11* in a wild type background by adding doxycycline to strain SC5314-*TETp*-*STE11* did not enhance biofilm formation. However, deletion of *TEC1*, which encodes the targeted transcription factor in the pheromone response pathway of white **a/a** cell biofilm formation, blocked **a**/α biofilm formation, as previously described [31]. (A) Quantitation of adhesion to a plastic surface after 16 h. (B) Images of adhesion of select strains to the plastic surface of wells after 16 h. (C) Biomass of biofilms formed after 48 h on a silicone elastomer surface. (D) ß-glucan released into the medium by 48 h biofilms. (E) Thickness of 48 h biofilms. (F) Cell density at the substratum (0 µm) and 20 µm above the substratum (20 µm) for 48 h biofilms of select strains. Data in panels A, C, D, and E are presented as mean ± standard deviation (error bar). Data are from eight measurements, two per biofilm preparation. Dox, doxycycline. Scale bar in panel H equals 100 µm.

Self-induction of a basic biofilm in an **a/a** white cell population, to which no opaque α/α cells are added, is mediated through the release of α-pheromone by minority **a/a** opaque cells produced by low frequency switching, in a paracrine-like fashion [16]. Addition of α/α opaque cells, which provide α-pheromone to a white **a/a** cell population, enhances biofilm formation by over 50% [16]–[18],[20]–[24]. α-pheromone stimulates white cell biofilm formation by binding to the α-pheromone receptor Ste2 on white **a/a** cells, which activates the MAP kinase signal transduction pathway. This pathway targets the transcription factor Tec1 [22]. The pheromone response pathway for **a/a** cells includes the pheromone receptor Ste2, the trimeric G protein complex, the MAP kinases Ste11, Hst7, and Cek1/Cek2, and the scaffold protein Cst5 and Tec1. Activation of white α/α cells involves the alternative **a**-pheromone and receptor Ste3 [16]. This relatively new pathway, which evolved in the ancestor of *C. albicans* and *C. dubliniensis* approximately 20 to 40 million years ago [37], borrowed intact the signals, receptors, trimeric G-protein complex, MAP kinase cascade, and scaffold protein from the pheromone response pathway involved in mating of opaque cells [24]. It seemed unlikely to us that the pathway for **a**/α biofilm formation, presumably a conserved and more ancient pathway than that of the white pheromone response pathway, would be regulated by genes derived from the mating process, given that these genes are suppressed in **a**/α cells by the **a**1-α2 corepressor [38],[39]. To exclude the MAP kinase pathway definitively, we assessed biofilm formation in the following deletion mutants that were generated in the **a/**α strain SC5314: *ste2/ste2*, the deletion mutant for the α-pheromone receptor; *ste11/ste11* and *hst7/hst7*, the deletion mutants for the MAP kinases Ste11 and Hst7; *cek1/cek1 cek2/cek2*, the double deletion mutant for the partially redundant MAP kinases Cek1 and Cek2 [20]; and *tec1/tec1*, the deletion mutant for the transcription factor Tec1 (Table S2) [22]. We also tested the **a**/α mutant *cph1/cph1*, the deletion mutant of the transcription factor Cph1targeted by the MAP kinase cascade in the opaque pheromone response pathway (Table S2) [26],[27]. Adhesion to a plastic surface after 16 h (Figure 2A,B), subsequent biofilm mass (Figure 2C), the level of ß-glucan released by the biofilm (Figure 2D), biofilm thickness (Figure 2E), and cell density at the substratum and 20 µm above the substratum in biofilms (Figure 2F) were similar for the **a**/α control strain SC5314 and the mutant derivatives *ste2/ste2*, *ste11/ste11*, *hst7/hst7*, *cek1/cek1 cek2/cek2*, and *cph1/cph1*. Since overexpression of *STE11* has been shown to activate the MAP kinase cascade in a pheromone- and receptor-independent fashion [22], we tested whether overexpression induced by doxycycline in strain SC5314-TETp-STE11 affected **a**/α biofilm formation. The characteristics of biofilms formed by cells in which *STE11* was overexpressed were similar to those of control SC5314 cells (Figure 2A through F). In the *tec1/tec1* mutant, however, adhesion was reduced by over 95% (Figure 2A,B), biofilm biomass by 90% (Figure 2C), ß-glucan release by 82% (Figure 2D), and biofilm thickness by 50% (Figure 2E). Cell density at the substratum and 20 µm above it in a biofilm was dramatically reduced (Figure 2F). These results demonstrate that the pheromone response pathway, from receptor through the MAP kinase pathway, is not involved in **a**/α biofilm formation, but the targeted transcription factor, Tec1, does play a role, as previously reported [28],[31]. Our results indicate, however, that Tec1 is regulated by a pathway other than the MAP kinase pathway in **a**/α biofilm formation.

### **a**/α Biofilms Are Regulated by the Ras1/cAMP Pathway

In **a**/α strains of *C. albicans*, the Ras1/cAMP pathway has been shown to be involved in the response of cells to a number of environmental cues. In particular, it has been demonstrated to play a role in the induction of filamentation [40]–[43], a major component of a mature biofilm [10],[11], in CO_2_, GlcNAc, and glucose induction of the white to opaque switch in *MTL*-homozygous cells [44], and in stationary phase and the starvation response [43]. We tested whether the Ras1/cAMP pathway also regulated **a**/α biofilm formation. The Ras1/cAMP pathway includes the following components: Ras1, a GTPase that activates adenylate cyclase [40],[42]; Cdc35, the only adenylate cyclase in *C. albicans* [45], which catalyzes the formation of cAMP; Pde2, a phosphodiesterase that acts as a negative regulator by hydrolyzing cAMP [46]; and Tpk1 and Tpk2, isoforms of cAMP-dependent protein kinase [47],[48]. The deletion mutants tested for biofilm formation were derivatives of the **a**/α strain SC5314 and included *ras1/ras1*, *cdc35/cdc35*, *pde2/pde2*, *tpk1/tpk1*, and *tpk2/tpk2* (Table S2). Complemented **a**/α derivatives of *ras1/ras1*, *pde2/pde2*, *tpk1/tpk1*, and *tpk2/tpk2* were generated with the respective wild type gene under the regulation of a tetracycline (doxycycline)-inducible promoter (Table S2) [49]. The complemented strains were *ras1/ras1-TETp-RAS1*, *pde2/pde2-TETp-PDE2*, *tpk1/tpk1-TETp*-*TPK1*, and *tpk2/tpk2-TETp-TPK2* (Table S2). A complemented derivative of *cdc35/cdc35* was generated by transformation with *CDC35* under the regulation of the *MET3* promoter, to generate *cdc35/cdc35-METp-CDC35* [42]. We also complemented *ras1/ras1* with a constitutively activated form of Ras1, Ras1V13 [40], to generate *ras1/ras1-TETp-RAS1V13* (Table S2).

**Figure 3:**
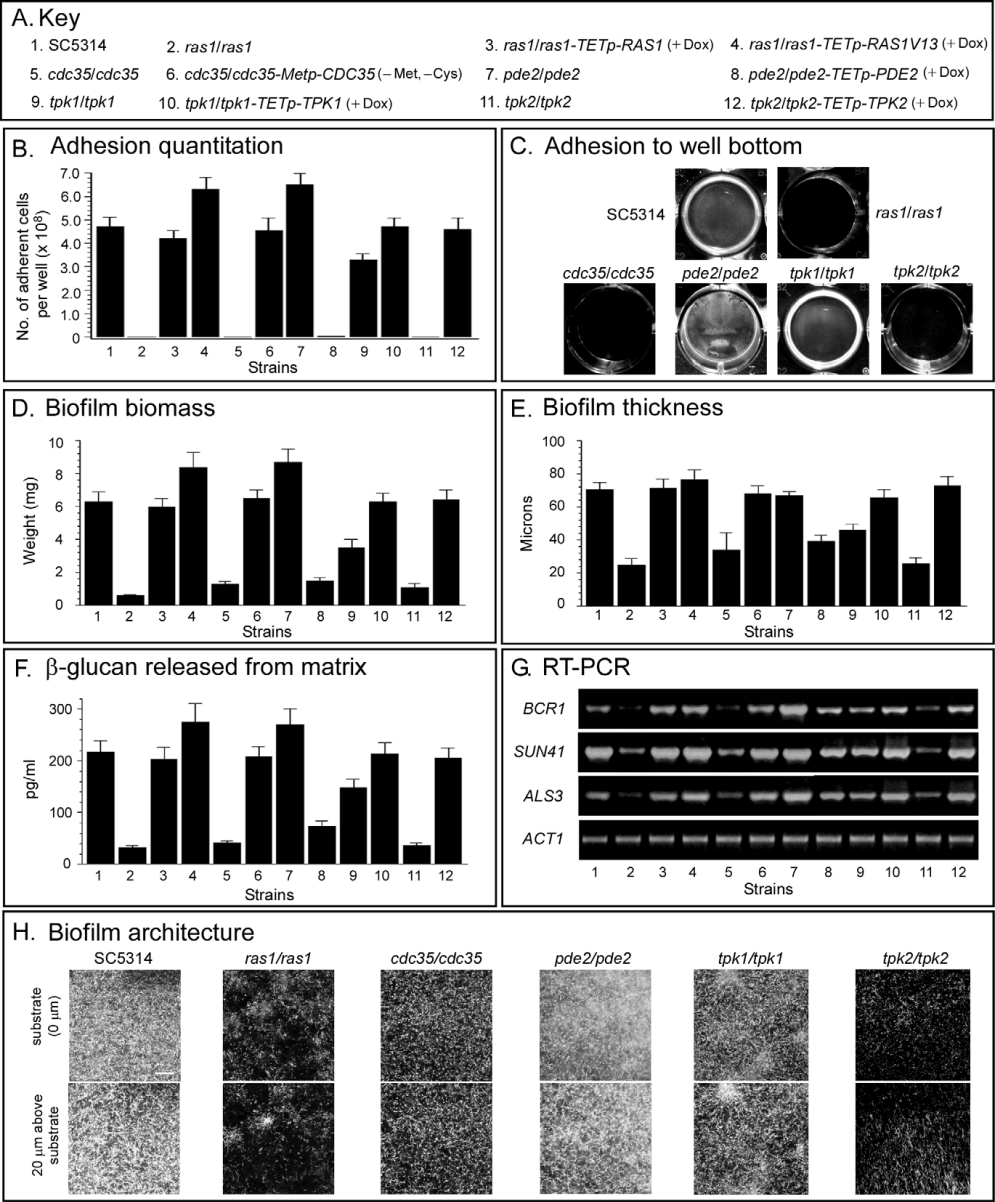
The Ras1/cAMP pathway regulates a/α biofilm formation. (A) Key to the mutants used in the analysis of the Ras1/cAMP pathway in panels B through H. SC5314 (**a**/α) was the parent strain (see Table S2 for origins and genotypes of mutants). (B) Quantitation of adhesion to a plastic surface after 16 h. (C) Images of adhesion of select strains to the plastic surface of wells after 16 h. (D) Biomass of biofilms formed on a silicone elastomer surface after 4 h. (E) Thickness of 48 h biofilms. (F) ß-glucan released into the media by 48 h biofilms. (G) The expression of three genes (*BCR1*, *SUN41*, *ALS3*), which are involved in **a**/α biofilm formation, assessed by reverse transcription-polymerase chain reaction (RT-PCR). Actin 1 expression is constitutive and used to assess loading. (H) Representative images of cell density at the substrate and 20 µm above the substratum for 48 h biofilms of select strains. Data in panels B, D, E, and F are presented as mean ± standard deviation (error bar). Data are from eight measurements, two per biofilm preparation. (−Met, −Cys), in the absence of methionine and cysteine, a condition that activates the methionine promoter (Metp); (+Dox), in the presence of doxycycline, which activates the tetracycline-inducible promoter (TETp). Scale bar in panel H equals 100 µm.

Deletion of *RAS1*, *CDC35*, and *TPK2* resulted in maximum reductions in all of the measured biofilm parameters. Adhesion to a plastic surface after 16 h (Figure 3B,C), subsequent biofilm biomass (Figure 3D), biofilm thickness (Figure 3E), the release of ß-glucan (Figure 3F), expression of *BCR1*, *SUN41*, and *ALS3* (Figure 3G), genes previously demonstrated to be up-regulated in **a**/α biofilms [28],[31],[50],[51], and cell density at the substrate and 20 µm above it (Figure 3H) were all dramatically reduced in the mutants *ras1/ras1*, *cdc35/cdc35*, and *tpk2/tpk2*, when compared to either the original parental strain SC5314 or to the relevant complemented strains. Deletion of *PDE2* resulted in biofilm parameters either equal to or slightly higher than those of the parental control strain, but overexpression in the complemented strain by the addition of doxycycline resulted in dramatically decreased or negligible biofilm parameters (Figure 3B through H), since Pde2 is a negative regulator of cAMP. Deletion of *TPK1* resulted in a partial reduction of biofilm parameters, whereas deletion of *TPK2* resulted in maximum reduction (Figure 3B through H). These latter results can be interpreted in two ways. First, Tpk1 and Tpk2 may perform different functions. Tpk2 may have a direct role, while Tpk1 may act as an enhancer. Alternatively, the two may be partially redundant, with Tpk2 able to compensate partially for the loss of Tpk1, but Tpk1 not able to partially compensate for the loss of Tpk2. Overexpression of *RAS1V13*, the activated form of *RAS1* [40], by addition of doxycycline to strain *ras1/ras1-TETp-RAS1V13*, resulted in an increase in select parameters, including adhesion (Figure 3B,C), biofilm biomass (Figure 3D), biofilm thickness (Figure 3E), and ß-glucan release (Figure 3F), to levels above that in control strain SC5314, as did the deletion of *PDE2*. Together, these results demonstrate that the Ras1/cAMP pathway regulates **a**/α biofilm formation, from the acquisition of adhesion and formation of the basal layer of yeast cells at the substrate, through maturation, resulting in hypha formation and the deposition of the biofilm matrix.

### **a/a** Biofilms Are Not Regulated by the Ras1/cAMP Pathway

The formation of white **a/a** biofilms has been demonstrated to be regulated by the MAP kinase pathway [16]–[18],[20]–[24]. To test whether the formation of *MTL*-homozygous biofilms was also regulated by the Ras1/cAMP pathway, **a/a** derivatives of the null mutants of two key components of the pathway, ras1/ras1 and tpk2/tpk2 (Table S2), were analyzed for several characteristics of biofilm formation. First, the two mutants were tested for α-pheromone-induced adhesion after 16 h of treatment on a plastic surface [15]. Cells of both **a/a** mutants *ras1/ras1* and *tpk2/tpk2* exhibited α-pheromone-induced adhesion similar to that of the **a/a** derivative of the parental strain SC5314 (Figure S1A). Biofilms formed by white cell populations of the two mutants on silicon elastomer in the absence of minority opaque α/α cells, a source of α-pheromone (unenhanced state; [15]), exhibited biomass (Figure S1B), released β-glucan level (Figure S1C), and cell density at the substrate and 20 µm above the substrate (Figure S1D), similar to that of the **a/a** derivative of the wild type strain SC5314. In the presence of 10% opaque α/α cells, a source of α-pheromone [15], biofilm biomass (Figure S1B), and released β-glucan (Figure S1C) was enhanced by approximately the same levels in *ras1/ras1* and *tpk2/tpk2* cells as in the **a/a** derivative of the wild type strain SC5314. Analysis of the cell types within the upper three-fourths of the **a/a** derivatives of *ras1/ras1* and *tpk2/tpk2* revealed vertically oriented hyphae (Figure S1F and S1H, respectively), absent from the **a**/α derivatives of *ras1/ras1* and *tpk2/tpk2* (Figure S1E and G, respectively). Viewing the cell types in **a/a** derivatives of both the mutant *ras1/ras1* and *tpk2/tpk2* revealed hypha formation in the upper three-fourths of the biofilm formed (Figure S1I and J, respectively). These results demonstrate that although the Ras1/cAMP pathway is essential for biofilm formation in **a**/α cells, including formation of vertically oriented hyphae and matrix in the upper portion of the biofilm, it is not essential for **a/a** biofilm formation. It should be noted that the hyphae formed in biofilms by the **a/a** derivatives of *ras1/ras1* and *tpk2/tpk2* were indistinguishable from those formed in biofilms of wild type **a/a** and **a**/α strains.

### Efg1, Tec1, and Bcr1 Function, in That Order, Downstream of the Ras1/cAMP Pathway

**Figure 4:**
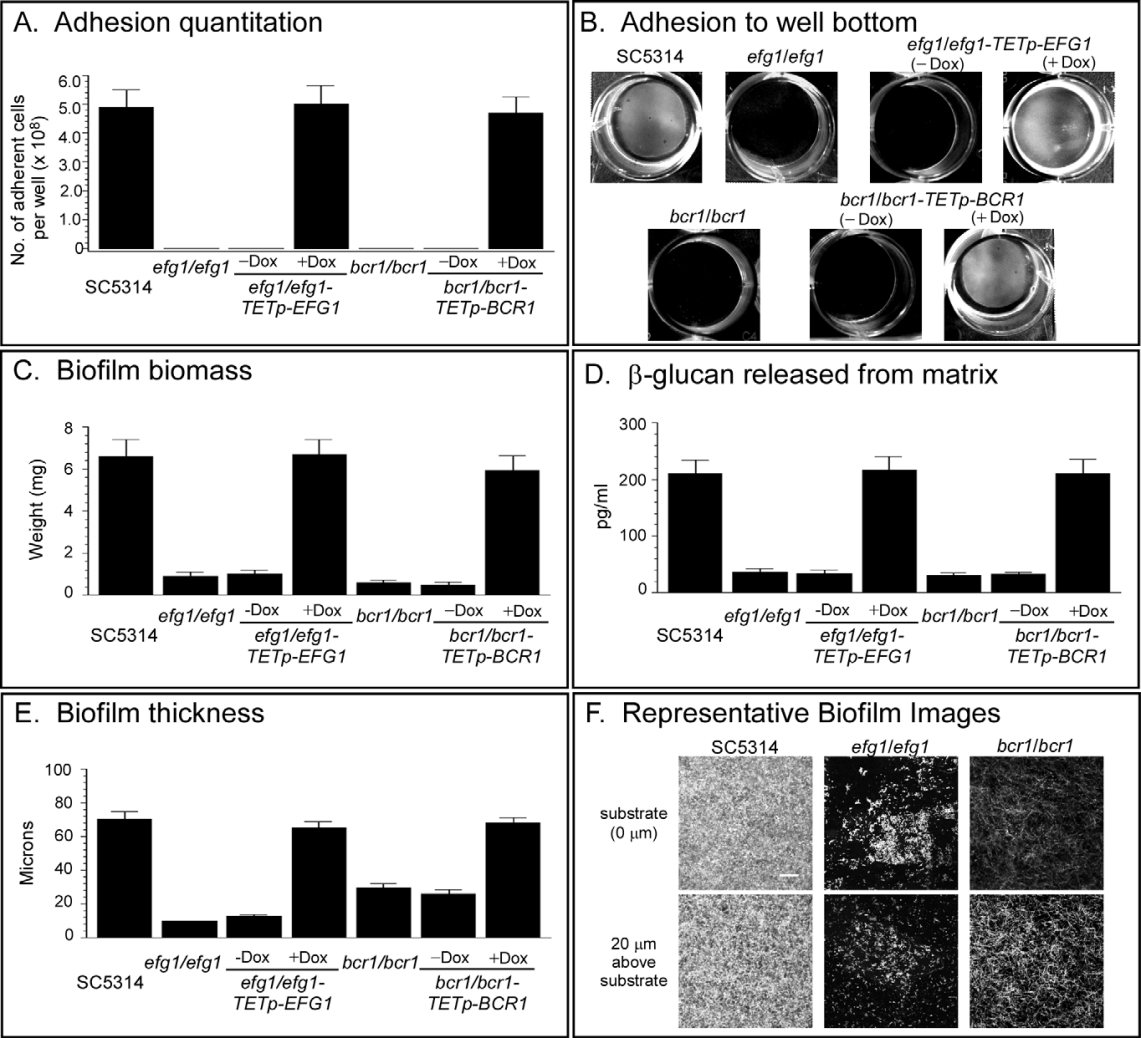
Deletion mutants of *EFG1* and *BCR1* in a/α cells have aberrant phenotypes similar to those of *ras1/ras1*, *cdc35/cdc35*, and *tpk2/tpk2*. See Table S2 for origins and genotype of strains. (A) Quantitation of adhesion to a plastic surface after 16 h. (B) Images of adhesion of select strains to the plastic surface of wells after 16 h. (C) Biomass of biofilms formed on an elastomer surface after 48 h. (D) ß-glucan released into the medium by 48 h biofilms. (E) Thickness of 48 h biofilms. (F) Representative images of cell density at the substratum and 20 µm above the substratum for 48 h biofilms of select strains. Data in panels A, C, D, and E are presented as mean ± standard deviation (error bars). Data are from eight measurements, two per biofilm preparation. (−Dox), in the absence of doxycycline; (+Dox), in the presence of doxycycline. Scale bar in panel F equals 100 µm.

**Figure 5:**
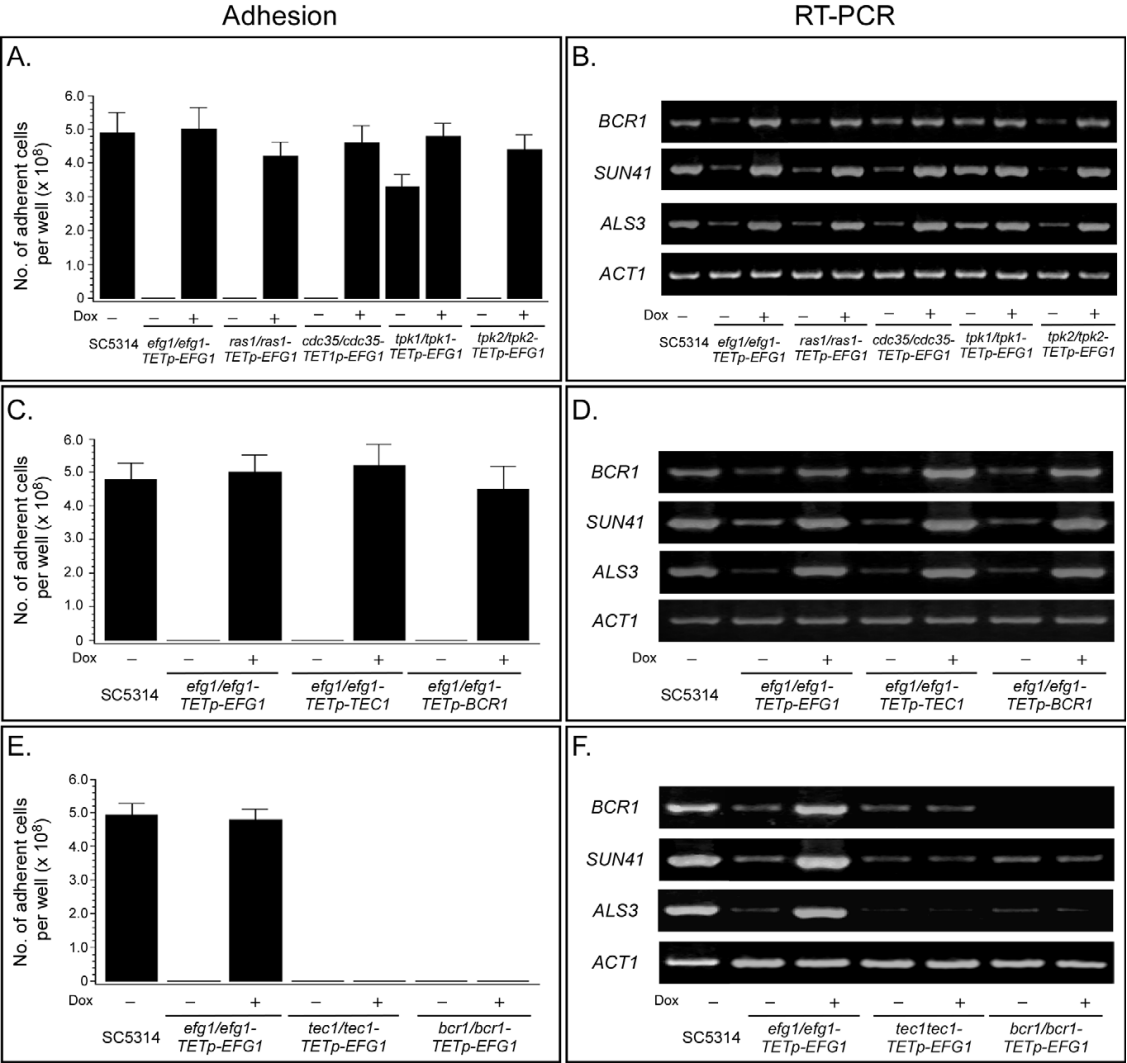
Efg1 functions downstream of the Ras1/cAMP pathway, but upstream of Tec1. To assess the order of function, wild type genes were placed under the regulation of a tetracycline (doxycycline)-inducable promoter in deletion mutant backgrounds, and tested for biofilm formation in the absence or presence of doxycycline. See Table S2 for origins and genotypes of mutants. (A) Adhesion when *EFG1* was overexpressed in the deletion mutants *efg1/efg1-TETp-EFG1*, *ras1/ras1-TETp-EFG1*, *cdc35/cdc35-TETp-EFG1*, *tpk1/tpk1-TETp-EFG1*, and *tpk2/tpk2-TETp-EFG1*. (B) Gene expression assessed by RT-PCR when *EFG1* was overexpressed. (C) Adhesion when *TEC1* or *BCR1* was overexpressed in mutants *efg1/efg1-TETp-TEC1* and *efg1/efg1-TETp-BCR1*. (D) Gene expression assessed by RT-PCR when *TEC1* or *BCR1* was overexpressed in the deletion *efg1/efg1* mutant background. (E) Adhesion when *EFG1* was overexpressed in the mutant *tec1/tec1-TETp-EFG1* or *bcr1/bcr1-TETp-EFG1* mutant. (F) Gene expression assessed by RT-PCR when *EFG1*was overexpressed in the *tec1/tec1* or *bcr1/bcr1* mutant background. Data in panels A, C, and E are presented as mean ± standard deviation for data from eight measurements, two per biofilm. Dox, doxycycline.

Having demonstrated that the Ras1/cAMP pathway regulates **a**/α biofilm formation, we examined the dependent relationship of this pathway with the three transcription factors that had previously been implicated in **a**/α biofilm formation. Efg1, a DNA binding protein [52] homologous to transcription factors involved in morphogenesis in a number of other fungal species [53]–[55], was shown by Ramage et al. [30] to be required for **a**/α biofilm formation. Bcr1, a C_2_H_2_ zinc finger protein known to bind to DNA as well as RNA and proteins [56], has also been shown to be necessary for the expression of *ALS3*, which encodes an adhesin that confers adherence in vitro and plays a role in **a**/α biofilm formation [28],[31]. Bcr1 has been demonstrated to function downstream of Tec1, the third transcription factor critical for **a**/α biofilm formation [28],[31]. Efg1 and Tec1 have been shown to function downstream of the Ras1/cAMP pathway in the regulation of hypha formation in **a**/α cells [57],[58], but Bcr1 has not been shown to be involved in hypha formation. Before assessing dependencies, we had to demonstrate that the aberrant phenotypes of the **a**/α mutants *efg1/efg1*, *bcr1/bcr1*, and *tec1/tec1* (Table S2) were similar to those of the **a**/α deletion mutant *ras1/ras1*, *cdc35/cdc35*, and *tpk2/tpk2*, using the same assays employed to characterize the latter. The **a**/α deletion mutants *efg1/efg1* and *bcr1/bcr1* exhibited dramatic reductions in adhesion (Figure 4A,B), biofilm biomass (Figure 4C), ß-glucan release (Figure 4D), biofilm thickness (Figure 4E), and cell density at the substrate and 20 µm above it (Figure 4F). These reductions were similar to those observed for the **a**/α mutants *ras1/ras1*, *cdc35/cdc35*, and *tpk2/tpk2* (Figure 3B,C,D,F,H). As already described, the **a**/α deletion mutant *tec1/tec1* also exhibited decreases similar to those of the **a**/α deletion mutants *ras1/ras1*, *cdc35/cdc35*, and *tpk2/tpk2* for adhesion (Figure 2A,B), biofilm mass (Figure 2C), released ß-glucan (Figure 2D), biofilm thickness (Figure 2E), and cell density (Figure 2F). The **a**/α mutant *tec1/tec1* also expressed reduced levels of *BCR1*, *SUN41*, and *ALS3* (see *tec1/tec1-TETp-EFG1*, minus doxycycline, in Figure 5F), as did the mutants *ras1/ras1*, *cdc35/cdc35*, and *tpk2/tpk2* (Figure 3G). The **a/**α mutants *efg1/efg1* and *bcr1/bcr1* also exhibited decreases in the expression of these three biofilm-related genes (see *efg1/efg1-TETp-EFG1* and *bcr1/bcr1-TETp-EFG1*, minus doxycycline, in Figure 5F).

Nobile and Mitchell [28] previously established that Bcr1 expression was regulated by Tec1. We therefore focused on the functional relationship of Efg1, first to the upstream Ras1/cAMP pathway, and then to Tec1 and Bcr1, using a promoter-regulated overexpression strategy. The **a**/α mutants *ras1/ras1*, *cdc35/cdc35*, and *tpk2/tpk2* were transformed with *EFG1* under the regulation of the tetracycline (doxycycline)-inducible promoter, *TETp*, generating strains *ras1/ras1-TETp-EFG1*, *cdc35/cdc35-TETp-EFG1*, and *tpk2/tpk2-TETp-EFG1* (Table S2). If normal **a**/α biofilms were formed when *EFG1* was overexpressed in these mutant backgrounds, then Efg1 functioned downstream of the Ras1/cAMP pathway. Two parameters were assayed, adhesion and up-regulation of biofilm genes. Overexpression of *EFG1* induced by the addition of the inducer doxycycline rescued the mutant phenotypes of *ras1/ras1*, *cdc35/cdc35*, and *tpk2/tpk2*, resulting in wild type levels of adhesion (Figure 5A) and wild type expression of *BCR1*, *SUN41*, and *ALS3* (Figure 5B). These results indicate that Efg1 functions downstream of the Ras1/cAMP pathway.

To determine the functional order of Efg1and the factors Tec1 and Bcr1, the **a**/α overexpression mutants *efg1/efg1-TETp-TEC1* and *efg1/efg1-TETp-BCR1* were generated (Table S2) and tested. If normal **a**/α biofilms were formed when *TEC1* or *BCR1* was overexpressed in an *efg1/efg1* background, then Tec1 and Bcr1 functioned downstream of Efg1. Overexpression of *TEC1* or *BCR1* by the addition of doxycycline rescued the *efg1/efg1* mutant phenotype for both adhesion and gene expression (Figure 5C and D, respectively), indicating that Tec1 and Bcr1 functioned downstream of Efg1. If true, then overexpressing *EFG1* in the mutants *tec1/tec1 or bcr1/bcr1* would not rescue either mutant phenotype. The **a**/α overexpression mutants *tec1/tec1-TETp-EFG1* and *bcr1/bcr1-TETp-EFG1* were, therefore, generated (Table S2) and tested. Overexpression of *EFG1* by the addition of doxycycline did not rescue either the *tec1/tec1* or *bcr1/bcr1* mutant phenotype (Figure 5E,F), supporting the conclusion that Tec1 and Bcr1 functioned downstream of Efg1. These results, together with those on the mutants of the Ras1/cAMP pathway and the observations of Nobile and Mitchell [28], define the following dependent pathway for the regulation of **a/**α biofilm formation: Ras1→Cdc35→cAMP (Pde2–**|**)→Tpk2(Tpk1)→Efg1→Tec1→Bcr1.

### Expression of Components in the Pathway Regulating **a**/α Biofilm Formation

To test whether components of the Ras1/cAMP pathway are selectively up-regulated during **a/**α, but not **a/a**, biofilm formation, the expression of *RAS1*, *TPK2*, *EFG1*, and *BCR1* were assayed by RT-PCR after 12 and 48 h either under planktonic conditions (P) or during biofilm development. All four genes were expressed under planktonic or biofilm conditions, after 12 or 48 h and in **a**/α or **a/a** cells (Figure S2). These results indicate that even though essential, *RAS1*, *TPK2*, *EFG1*, and *BCR1* are not selectively up-regulated during **a**/α biofilm formation. All are constitutively expressed.

### Overexpression of *BCR1* Only Partially Rescues the *tec1/tec1* Mutant Phenotypes in **a**/α Cells

Nobile et al. [31] demonstrated that overexpression of *BCR1* in the **a**/α mutant *tec1/tec1* partially rescued the defect in biofilm biomass, but not hypha formation within the biomass, perhaps because Tec1 is involved in regulating hypha formation independently of biofilm formation [59]. If the transcription factor Bcr1 alone regulates biofilm genes and Tec1 regulates only Bcr1 expression in the **a**/α biofilm pathway, then overexpression of Bcr1 in a *tec1/tec1* background might rescue the adhesion defect, which is an early developmental stage in the maturation of biofilms preceding hypha formation and matrix deposition in biofilm development. We, therefore, tested whether overexpression of *BCR1* in the mutant *tec1/tec1-TETp-BCR1* restored adhesion. Overexpression induced by the addition of doxycycline partially restored adhesion to a level approximately two-thirds that of the parental strain SC5314 (Figure S3), a result similar to that of Nobile et al. [31]. These results suggest that although Tec1 is upstream of Bcr1, both Bcr1 and Tec1 regulate downstream genes early in **a**/α biofilm formation.

### Efg1 Is Regulated by Phosphorylation

**Figure 6:**
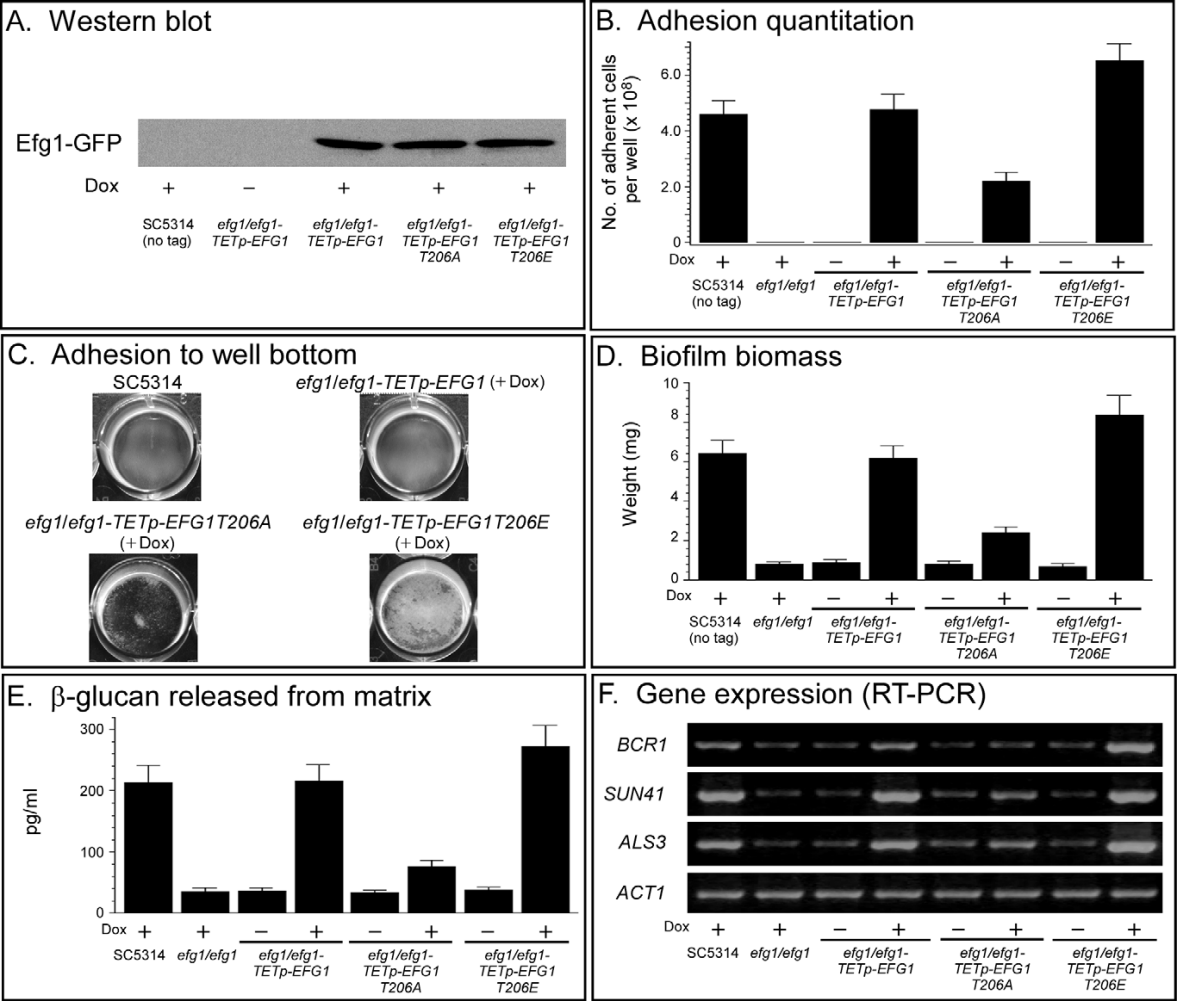
The activity of Efg1 is regulated by phosphorylation of a threonine, at amino acid 206. The deletion mutant *efg1/efg1* was transformed with *EFG1T206A*, in which threonine was replaced with alanine, thus mimicking the constitutively unphosphorylated state, or with *EFG1T206E*, in which threonine was replaced with glutamic acid, thus mimicking the constitutively phosphorylated state. Both *EFG1T206A* and *EFG1T206E* were placed under the regulation of the tetracycline (doxycycline)-inducible promoter to generate strains *efg1/efg1-TETp-EFG1T206A* and *efg1/efg1-TETp-EFG1T206E.* The transformation constructs were tagged with GFP (see Table S2 for genotypes). Efg1 had previously been shown to be regulated by phosphorylation at threonine 206 [60]. (A) Levels of expression of Efg1 measured by western blot staining with anti-GFP antibody. (B) Quantitation of adhesion to a plastic surface after 16 h. (C) Images of adhesion by selective strains to the plastic surface after 16 h. (D) Biomass of biofilms formed on a silicone elastomer surface after 48 h. (E) ß-glucan released into the medium by 48 h biofilms. (F) Gene expression using RT-PCR of 48 h biofilms. Data in panels B, D, and E are presented as the means ± standard deviation. Data are from eight measurements, two per biofilm preparation.

Bockmühl and Ernst [60] previously demonstrated that the role of Efg1 in filamentation was regulated through phosphorylation of a single threonine residue at amino acid 206. Since Efg1 is the first protein we identified downstream of the cAMP-dependent kinase, Tpk2 (Tpk1), in the pathway regulating **a**/α biofilm formation, we tested whether Efg1 had to be phosphorylated at this site in order to function in the regulation of **a**/α biofilm formation. This represented the only identifiable cAMP-dependent kinase phosphorylation site in the deduced amino acid sequence. The deletion mutant *efg1/efg1* was transformed with native *EFG1*, the derivative *EFG1T206A*, which contains alanine instead of threonine at amino acid 206, thus mimicking the constitutively unphosphorylated state, and the derivative *EFG1T206E*, which contains glutamic acid at amino acid 206, thus mimicking the constitutively phosphorylated state. The generated strains were *efg1/efg1-TETp-EFG1*, *efg1/efg1-TETp-EFG1T206A*, and *efg1/efg1-TETp-EFG1T206E*, all tagged with GFP (Table S2). Western blot analysis using anti-GFP antibody revealed that in the three respective strains, the level of Efg1, Efg1T206A, and Efg1T206E were similarly up-regulated by doxycycline (Figure 6A). Overexpression of wild type *EFG1* in *efg1/efg1-TETp-EFG1* reestablished wild type levels of adhesion (Figure 6B,C), biofilm biomass (Figure 6D), released ß-glucan (Figure 6E), and gene expression (Figure 6F). Overexpression of *EFG1T206A*, in *efg1/efg1-TETp-EFG1T206A*, resulted in partial increases in adhesion (Figure 6B,C), biofilm biomass (Figure 6D), released ß-glucan (Figure 6E), and gene expression (Figure 6F). In each case the increase was roughly a third of that achieved by overexpression of wild type *EFG1*. In marked contrast, overexpression of *EFG1T206E* resulted in increases in adhesion (Figure 6B,C), biofilm biomass (Figure 6D), release of ß-glucan (Figure 6E), and gene expression (Figure 6F) that were at least as great as that achieved when wild type *EFG1* was overexpressed. Together these results indicate that Efg1 is activated at least in part in the **a**/α biofilm pathway through phosphorylation.

### *MTL*-Homozygous Biofilms Are Not Regulated by Bcr1

The **a/a** mutant bcr1/bcr1 exhibited adhesion levels after 16 h of α-pheromone treatment similar to that of wild type **a/a** cells (Figure S1A). The subsequent characteristics of the biofilms formed by the **a/a** mutant after 48 h (biomass, β-glucan release, and cell density at the substrate and 20 µm above it) were also similar to the those of biofilms formed by the **a/a** wild type strain in the absence (unenhanced) or presence (enhanced) of minority opaque α/α cells (Figure S1B, C, and D, respectively). These results indicate that even though *BCR1* is expressed at similar levels during **a**/α and **a/a** biofilm formation, *BCR1* is essential only for the former.

### Overexpression of *BCR1* Confers Impermeability to **a/a** Biofilms

Even though *BCR1* transcript levels were similar during **a**/α and **a/a** biofilm formation (Figure S2), *BCR1* was essential only for the former. We, therefore, expected to find that deleting the gene or overexpressing it would have no effect on the permeability of **a/a** biofilms. As expected, deleting *BCR1* in the **a/a** strain P37005 had no further effect on the high degree of biofilm permeability (Figure S4A,B). However, overexpressing *BCR1* in strain **a/a** P37005-TETp-BCR1 (Table S2), by adding doxycycline, resulted in a dramatic decrease in the permeability of SYPRO Ruby (Figure S4C through E). The decrease was a function of the level of overexpression. Permeability in parental P37005 biofilms was 100% (*N* = 9) in 0 µg/ml of doxycycline, 34.5±4.6% (*N* = 9) in 25 µg/ml of doxycycline, and 21.9±4.0% (*N* = 9) in 50 µg per ml of doxycycline (Figure S4C, D, and E, respectively). Neither deletion of *BCR1*, in the *bcr1/bcr1* **a/a** mutant, nor overexpression in strain P37005-*TETp*-*BCR1*, affected biofilm thickness (Figure S4A through E) or in the level of β-glucan released into the medium (Figure S4F). These results suggest that in **a**/α cells, the constitutive level of expression is sufficient to affect impermeability, but in **a/a** cells, that constitutive level is insufficient. Furthermore, Bcr1-induced impermeability does not appear to involve increased β-glucan synthesis.

## Discussion

### Permeability, Drug Susceptibility, and Penetrance

The formation of an **a**/α biofilm and that of a unisexual *MTL*-homozygous (**a/a** or α/α) white cell biofilm follow roughly the same developmental stages, which include the formation of a basal layer of yeast phase cells on an adherent surface, the formation and extension of vertically oriented hyphae from the basal layer, and the deposition of a polymolecular extracellular matrix. **a**/α biofilms are approximately 30% thicker than unisexual **a/a** or α/α biofilms, but the thickness of the latter can be enhanced approximately to that of **a**/α biofilms by adding a minority of opaque cells of opposite mating type, a source of pheromone [15]. Here, we tested the hypotheses that although superficially similar, the function and regulation of the two basic types of biofilms, **a**/α and unenhanced **a/a** or α/α, differ. We have presumed that because the *MTL* configuration of a majority of strains (∼90%) causing commensal colonization and infection in nature is **a**/α [4]–[7], a similar majority of biofilms formed in hosts are **a**/α. Biofilms formed by **a/**α cells appear to play the traditional role of microbial biofilms, providing a controlled, protective multicellular environment, resistant to environmental challenges [10]–[14],[61]–[63]. We therefore expected to find, as we did, that **a**/α biofilms formed on silicon elastomers were impermeable to molecules in the size range of antifungals and antibodies. A number of prior studies revealed that *C. albicans* **a**/α biofilms were resistant to antifungals [61],[64],[65] and that resistance late in biofilm development was not dependent upon the up-regulation of efflux pumps [65]. The impermeability that we have found to Dead Red, SYPRO Ruby, and ConA is very likely the basis for the general resistance to fluconazole and other antifungals [66]. Given this general impermeability characteristic, it was not surprising to find that **a**/α biofilms were also highly resistant to penetration by human polymorphonuclear leukocytes. Katragkou et al. [67] had previously performed a similar experiment in which they overlayed biofilms with monocytes and observed progressive penetration to the middle layer after 22 h. However, they used a monocyte/*C. albicans* cell ratio of 1∶1 to 10∶1, whereas we used a ratio of 1∶3,000. It is, therefore, difficult to compare their results with ours. Impenetrability by white blood cells of bacterial biofilms has also been observed. Bjarnsholt et al. [68] demonstrated that PMNs did not penetrate biofilms formed by *Pseudomonas aeruginosa* and hypothesized that this impenetrability might be the reason for chronic ulcers in the legs and feet of diabetics [69].

Biofilms formed by white **a/a** or α/α cells have been shown in vitro to facilitate the mating process between seeded minority opaque cells of opposite mating type by protecting gradients of pheromones that direct chemotropism [15],[19]. Given that pheromones, with a molecular weight of approximately 1.6 kDa, must readily diffuse through an *MTL*-homozygous biofilm, we considered the possibility that in contrast to **a**/α biofilms, **a/a** and α/α biofilms would be more permeable to low molecular weight molecules. Moreover, since chemotropism in an *MTL*-homozygous biofilm involves extension and penetration of long conjugation tubes with diameters of up to 1 µm [15], we also considered the possibility that human PMNs penetrated **a/a** and α/α biofilms more readily than they did **a**/α biofilms. Our results support these predictions. Our results are consistent with the hypothesis that the main role of **a**/α biofilms is to provide an impermeable, impenetrable environment that protects cells from environmental challenges during commensalism and infection, but that the role of white **a/a** and α/α biofilms may be to provide a permeable, penetrable multicellular environment that functions, at least in part, to support chemotropism and fusion of opaque cells of opposite mating types in the mating process. Whatever the role proves to be for *MTL*-homozygous biofilms, our results clearly demonstrate for the first time that *C. albicans* forms two morphologically similar but functionally distinct biofilms, depending upon the configuration of the *MTL* locus.

### Alternative Regulation of **a**/α and **a/a** Biofilms

**Figure 7:**
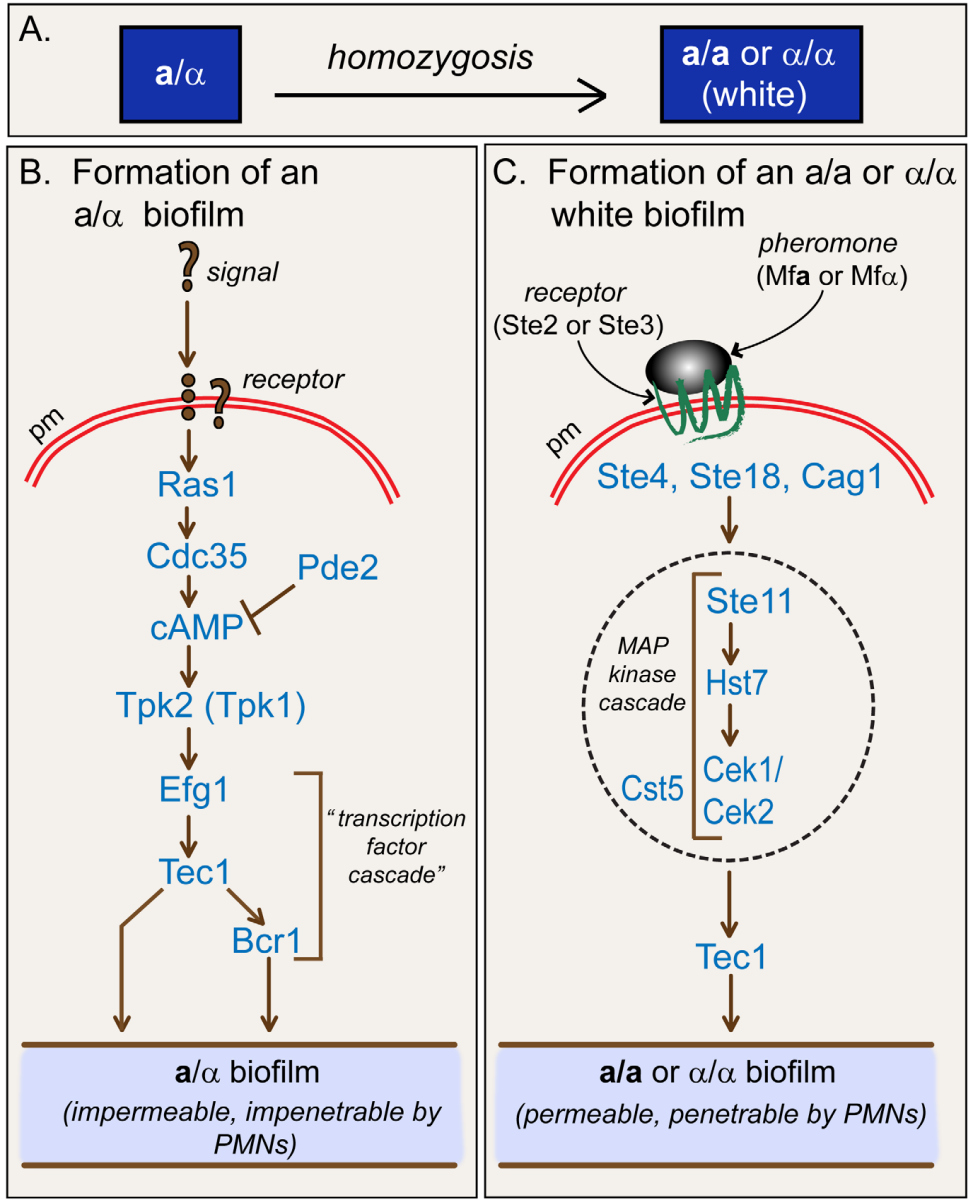
Alternative models for the regulation of a/α and a/a or α/α biofilms. (A) **a**/α cells undergo homozygous to **a/a** or α/α. (B) Regulation of **a**/α biofilm formation. Question marks refer to unknown signal and receptor of this signal transduction pathway. (C) Regulation of **a/a** (or α/α) biofilm formation. Regulation of α/α biofilms is assumed to be similar to that of **a/a** biofilms, for which we have presented evidence. Similar permeability and penetrability characteristics have been demonstrated for **a/a** and α/α biofilms. pm, plasma membrane.

For *C. albicans* to form an **a/a** or α/α white cell biofilm, **a**/α cells must first undergo *MTL*-homozygosis to the **a/a** or α/α *MTL* configuration (Figure 7A), releasing the switching system from **a**1-α2 repression [4],[8]. White cells of a single mating type (i.e., **a/a** or α/α) are then activated through mating type-nonspecific release of pheromone in a paracrine signaling system [16],[17],[18] to form an **a/a** or α/α biofilm. The α- or **a**-pheromone signal is transduced in white cells by a pathway (Figure 7C) that includes the pheromone receptors Ste2 or Ste3, respectively, the trimeric G-protein complex Cag1, Ste4, and Ste18, the MAP kinase cascade Ste11, Hst7, and Cek1/Cek2, and the MAP kinase scaffold Cst5 (Figure 7C) [20],[23],[24]. This represents the same upper portion of the pathway that regulates the opaque cell pheromone response in the mating process [24]. In the formation of a white **a/a** biofilm, the MAP kinase pathway activates the transcription factor Tec1 through phosphorylation [22]. Tec1 then up-regulates biofilm-related genes by binding to the AT-rich *cis*-acting sequence WPRE in the promoters of those genes [18]. Here we have demonstrated by mutational analysis that although this pathway is essential for the formation of a white *MTL*-homozygous biofilm (Figure 7B), it plays no role in the formation of an **a**/α biofilm.

Instead, we have found that **a**/α biofilms are regulated by the following pathway: Ras1→Cdc35→cAMP(Pde2–**|**)→Tpk2(Tpk1)→Efg1→Tec1→Bcr1 (Figure 7A). Our results suggest that Tpk2, the cAMP dependent kinase at the end of the Ras1/cAMP portion of the pathway, is essential for **a**/α biofilm formation, and directly or indirectly activates Efg1 through phosphorylation of threonine 206, the only protein kinase A motif identifiable in the protein [60],[70]. Tpk1, although not essential, appears to play a facilitating role, apparently by increasing the efficiency of the response, but is unable to compensate for the deletion of Tpk2. Except for the role of Bcr1, this represents the same pathway that regulates hypha formation in **a**/α cells [42]–[48]. It had previously been demonstrated that the transcription factors Efg1, Tec1, and Bcr1 were necessary for normal **a**/α biofilm development, and that Tec1 regulated *BCR1* expression [31]. Since these last three components of the **a**/α biofilm pathway are transcription factors, the dependent sequence Efg1→Tec1→Bcr1 may be considered a transcription factor cascade, Efg1 activating Tec1, and Tec1 in turn activating Bcr1. Given that Bcr1 is at the end of this dependent pathway, one might conclude that it is ultimately responsible for up-regulating all **a**/α biofilm genes. However, overexpression of *BCR1* in a *tec1/tec1* background in **a**/α cells did not fully rescue the mutant aberrant adhesion phenotype of *tec1/tec1*, a result similar to that obtained by Nobile et al. [31], suggesting that Tec1 directly regulates Bcr1, but both Tec1 and Bcr1 regulate genes that contribute to **a**/α biofilm formation, as modeled in Figure 7B.

By mutational analysis, we have also shown that deletion of two major components of the Ras1/cAMP pathway, *RAS1* and *TPK2*, has no measurable effect on the formation of **a/a** biofilms. Deletion of *BCR1* was further shown to have no effect on the high level of permeability to SYPRO Ruby. We have also shown that even though *RAS1* and *TPK2* are expressed constitutively at similar levels in **a**/α and **a/a** cells, they only play a role in the formation of an **a**/α biofilm. Therefore, **a**/α biofilms are regulated by the Ras1/cAMP pathway, but not the MAP kinase pathway, while *MTL*-homozygous (**a/a** and α/α) biofilms are regulated by the MAP kinase pathway, but not the Ras1/cAMP pathway (Figure 7). It should be kept in mind, however, that identification of these specific pathways does not exclude the existence of additional alternative or common pathways regulating **a**/α and *MTL*-homozygous biofilms.

Interestingly we have found that even though *BCR1* is not essential for **a/a** biofilm formation, it is expressed at a level similar to that in **a**/α cells. Furthermore, overexpression in **a/a** cells results in a decrease in permeability that mimics the impermeability characteristic of **a**/α biofilms. It is not immediately clear why overexpression of *BCR1* in **a/a** cells, which already express *BCR1* at a constitutive level, confers this impermeability characteristic. Together, these results suggest a post-transcriptional modification process that represses *BCR1*function, but which cannot accommodate abnormally higher concentrations of *BCR1* when overexpressed. Regardless of the reason for this apparent paradox, our results suggest that Bcr1 is involved in the regulation of genes involved in **a/**α biofilm impermeability.

### The Regulation of Hypha Formation in Alternative Biofilms

The Ras1/cAMP pathway plays a major role in the regulation of hypha formation in **a**/α cells [40]–[43],[71],[72]. Deletion of components of this pathway in **a**/α cells impairs, delays, or blocks the formation of hyphae. This pathway was then implicated in the establishment of stationary phase and the starvation response in **a/**α cells [43] and CO_2_, GlcNAc, and glucose induction of the white to opaque switch in *MTL*-homozygous cells [44]. Here we demonstrate that this same pathway plays a key regulatory role in **a**/α biofilm formation, which includes early adhesion and subsequent hypha and matrix formation during biofilm maturation. We also demonstrate that the Ras1/cAMP pathway does not play a similar role in **a/a** biofilm formation. In **a**/α cells, both the mutants *ras1/ras1* and *tpk2/tpk2* do not undergo an increase in adhesion, do not form a uniform yeast basal cell layer early in biofilm formation, and do not form hyphae vertically oriented in an extracellular matrix later in biofilm formation. In **a/a** cells, however, the same mutants, *ras1/ras1* and *tpk2/tpk2*, form normal biofilms that include vertically oriented hyphae in a matrix. The hyphae formed in these mutants are indistinguishable from those formed by wild type **a/a** cells and by wild type **a**/α cells during biofilm formation. These results indicate that during **a/**α biofilm formation, the Ras1/cAMP pathway is involved in the regulation of early as we well as late events, including hypha and matrix formation, but in **a/a** biofilm formation, this pathway is not involved.

### **a/a** Biofilms May Have Evolved from **a**/α Biofilms

The major pathogenic species of the *Candida* clade, including *C. albicans*, *C. parapsilosis*, and *C. tropicalis*, form biofilms on silicone elastomers [25]. Although differences have been noted between these biofilms, including cellular composition, matrix, and drug susceptibility [10]–[14],[25], the capacity to form a biofilm appears to be a general characteristic that, like the mating process [24],[73]–[75], appears to have been conserved throughout the evolution of this clade. Identifying the signal transduction pathway controlling **a**/α biofilm formation in *C. albicans* should provide a contextual framework for testing whether biofilm formation in all members of this clade are similarly regulated.

The results we have obtained here also support the hypothesis that has been proposed for the evolution of the regulatory pathway for *MTL*-homozygous biofilms [22],[23]. Because *C. albicans* [76] and the closely related species *Candida dubliniensis* [77] are the only two members of the *Candida* clade that have been found to undergo white-opaque switching and the white cell pheromone response, these developmental programs most likely evolved in the ancestor to the two species, approximately 40 million years ago [19],[37]. The upstream portion, from signal and receptor through the MAP kinase cascade, appears to have been derived completely intact from the conserved upstream portion of the pheromone response pathway for mating [20],[24]. We must now consider the possibility that the downstream portion, which includes the targeted transcription factor Tec1 and the genes it activates, may have been derived from the downstream portion of the conserved pathway for **a**/α biofilm formation [21],[23]. The evolutionary transition, however, could not have been an intact transfer, as appears to be the case for the upper portion of the pathway. Two major changes would have had to have taken place. First, Tec1, which is under the regulation of Efg1 in the **a**/α pathway, would have had to come under the regulation of Cek2 (Cek1). Second, Tec1 would have had to lose the capacity to activate genes through Bcr1, as it does in **a**/α biofilm formation. If this hypothesis is correct, then **a**/α biofilms would benefit from the genes activated by both Tec1 and Bcr1, whereas **a/a** and α/α biofilms would benefit solely from genes activated by Tec1. The difference, therefore, would be the selective expression of *BCR1*-activated genes in **a**/α biofilms, and could explain the differences between the two types of biofilms in regard to permeability, drug susceptibility, and white blood cell penetrance. We have therefore shown that *C. albicans* forms two morphologically similar but functionally distinct biofilms depending upon the configuration of the *MTL* locus. The **a**/α biofilm appears to represent the conserved form functioning traditionally as an impermeable impenetrable barrier to host challenges. The **a/a** or α/α biofilm, in contrast, appear to represent a recently evolved form, both permeable and penetrable, which may serve functions related to the facilitation of mating.

## Materials and Methods

### Strains and Media

The genotype and origins of all parental, mutant, and complemented strains employed in this work are listed in Tables S1 and S2. Strains were standardly grown at 25°C on agar plates or in liquid either containing modified Lee's medium [78],[79] or YPD medium [80] prior to experimental use. Deletion mutants and complemented strains were generated according to methods previously described [17],[18],[20], which were adapted from Reuss et al. [81]. The primers used for the generation of mutants and complemented strains are presented in Table S3. In select cases, to obtain **a/a** or α/α strains from **a**/α strains, cells were treated with L-sorbose [82], plated, and opaque sectors (**a/a** or α/α derivatives) were selected and analyzed by PCR for the configuration of the *MTL* locus.

### Gene Overexpression Strategy

A tetracycline-inducible or a methionine-, cysteine-repressible system was employed for overexpression studies. For tetracycline (doxycycline) inducibility, the plasmid pNIM1 [49] was used, which harbors a *GFP* coding region and the tetracycline-regulated promoter *TETp*. The ORF of a gene was amplified by PCR with the primers listed in Table S3. The amplified fragments were subcloned into the plasmid pNIM1at the SalI site, to generate derivatives under the control of *TETp*. The *GFP* gene was fused in-frame to the 3̀ end of the ORF and the correct orientation confirmed by sequencing. The *TETp*-gene *GFP* fusion plasmids were digested with ApaI plus SacII and transformed into either wild-type or mutant strains. The constructs were targeted to the *ADH1* locus. Activation of gene transcription by doxycycline was verified by RT-PCR analysis prior to experimentation. In the case of *CDC35* overexpression, the *MET3* repression-based plasmid pK75.2 [83] was used instead. The *CDC35* ORF, derived by PCR with primers listed in Table S3, was digested with SbfI and ligated at the PstI site of pK75.2 to derive the overexpression plasmid.

### Measurement of Biofilm Formation

Methods for measuring adhesion [15], biofilm biomass [23], release of β-glucan [18],[84], biofilm thickness [20], and cell density [23] have been described previously in detail. For adhesion assays, cells were incubated for 16 h on a plastic surface, and for the remaining assays, cells were incubated for 48 h on a silicone elastomer surface [15],[20].

### Constitutively Expressed GFP Strains, Dye Permeability, and PMN Penetration

To generate strains constitutively expressing GFP, the promoter of the actin gene (*ACT1*) was amplified as a 1 kb fragment upstream of the ATG start codon using primers described in Table S3. The PCR product was digested with SalI and subcloned into the SalI-digested, dephosphorylated plasmid pNIM1. The plasmid was linearized by digestion with ApaI and SacII, and transformed into isogenic **a**/α, **a/a**, and α/α derivatives of strains P37039, P37037, and SC5314. The fluorescent strains are described in Table S1.

To determine dye penetration, 48 h biofilms were overlaid with a solution containing Film Tracer SYPRO Ruby dye (Invitrogen) for 30 min prior to live confocal imaging, as previously described [23]. Simultaneous GFP (argon laser 488 excitation/515 emission) and SYPRO Ruby (argon laser 457 excitation/610 emission) fluorescent images were acquired as a z-series at 1 µm intervals through 100 µm. After *z*-series acquisition, a *z*-image through the image stack, perpendicular to the substrate, was generated to assess the limits of dye penetration.

To assess human polymorphonuclear leukocyte (PMN) penetrance, PMNs were purified from venous blood as previously described [85]. The purified cells were suspended in Hank's Balanced Salt Solution (Gibco-BRL, Gaithersburg, MD) at a final concentration of 1.5×10^6^ cells/ml, labeled with 1 µM Vybrant CM-DiI (Molecular Probes, Invitrogen) for 5 min and washed with RPMI medium. Twenty µl of PMNs were overlaid on 48 h biofilms and incubated for 3 h at 37°C in 5% CO_2_. Fluorescent images were acquired as above, but *z*-slices were acquired.

To assess the effects of fluconazole (Sigma, St. Louis) on cell viability, 1.5 ml of medium were removed from each well of 48 h biofilm cultures [15] and replaced with 1.5 ml of fresh RPM1 culture medium. Fluconazole was added to a final concentration of 25 µg per ml. Biofilms were returned to the rocker for an additional 24 h. Each silicone elastomer disc with attached biofilm was briefly rinsed with PBS and placed in a 1.5 ml Eppendorf tube containing 1 ml of Dulbecco's PBS without cations, supplemented with 20 mM EDTA. The biofilms were incubated for 5 min and vigorously vortexed to remove the biofilm from the elastomer disks. The cleared elastomer disk was removed and the efficiency of biofilm assessed microscopically. The biofilm cells were pelleted and resuspended in PBS containing 0.25 µM Dead Red, a dead cell nuclear stain (Invitrogen). The total number of cells in a microscopic field (20× objective) was counted and the number of labeled nuclei in the same field determined by excitation at 543 nm. Three fields were counted for duplicate biofilms of each strain.

### RT-PCR

Treatment with a 0.05% trypsin-EDTA solution (Invitrogen) released biofilms from the substrate. Total RNA was extracted using the RNeasy Mini Kit (Qiagen, Valencia, CA). Reverse transcription-Polymerase Chain Reaction (RT-PCR) was used to analyze gene expression levels according to methods previously described [16],[23],[31],[86]. The primers used are listed in Table S4.

### Site-Directed Mutagenesis

Site-directed mutagenesis was performed for *EFG1* according to methods previously described [23]. In brief, the 5′ and 3′ portions of the *EFG1* gene flanking the site to be mutated were amplified by PCR using primers listed in Table S3. The two DNA fragments were then fused together by PCR using 5′ and 3′ primers. The resulting mutated ORF was digested with SalI and subcloned into the SalI-digested, dephosphorylated plasmid pNIM1. GFP was fused in-frame to the C-terminal region of the mutated versions of *EFG1* and its mutant derivatives. The plasmid was then digested with ApaI and SacII and transformed into the homozygous deletion mutant *efg1/efg1*. The derived point mutants were verified by PCR and sequencing.

### Western Blotting

Western blots were performed according to methods previously described [23],[44].

## Supporting Information

Figure S1 Ras1, Tpk2, and Bcr1 play no measurable role in **a**/**a** biofilm formation. Parental and mutant **a**/**a** strains are generated from **a**/**a** strains (see Table S2 for genotype origins and references). Methods can be found in Materials and Methods. Scale bar equals 100 µm. Note that in panels E and G, the use of a projection image obscures the true patchiness of the cell layers on the substratum.
(TIF)

Figure S2 Expression of *RAS1*, *TPK2*, *EFG1*, and *BCR1* in biofilms formed by **a**/**a** strain P37005 and **a**/**a** strain SC5314 under planktonic growth (P) and biofilm formation (BF) after 12 and 48 h of development. Methods can be found in Materials and Methods. *TDH* expression is known to be constitutive.
(TIF)

Figure S3 Overexpression of *BCR1* in a *tec1/tec1* mutant in **a**/**a** cells only partially rescues the defective adhesion phenotype.
(TIF)

Figure S4 Overexpression of *BCR1* in the **a**/**a** strain P37005 results in an increase in impermeability to Sypro Ruby. Thickness (Thick.) and permeability (Perm.) were quantitated. (A through E) Sypro Ruby staining of 48-h, live biofilms. (F) β-glucan released into medium. Scale bar equals 100 µm.
(TIF)

Table S1 GFP-tagged strains used for biofilm dye permeability, cell penetration, and fluconazole susceptibility.
(DOC)

Table S2 Strains used in mutant studies.
(DOCX)

Table S3 Oligonucleotides used for mutant construction.
(DOC)

Table S4 Primer used for RT-PCR.
(DOCX)

## Abbreviations

LSCM: laser scanning confocal microscopy
PMN: polymorphonuclear leukocyte
RT-PCR: Reverse Transcription–Polymerase Chain Reaction
