# Supplementary figures and images for: Alternative Mating Type Configurations (a/α versus a/a or α/α) of Candida albicans Result in Alternative Biofilms Regulated by Different Pathways
Source: PLoS Biol. 2011 Aug 2;9(8):e1001117. doi: 10.1371/journal.pbio.1001117 (PMC3149048; doi:10.1371/journal.pbio.1001117)

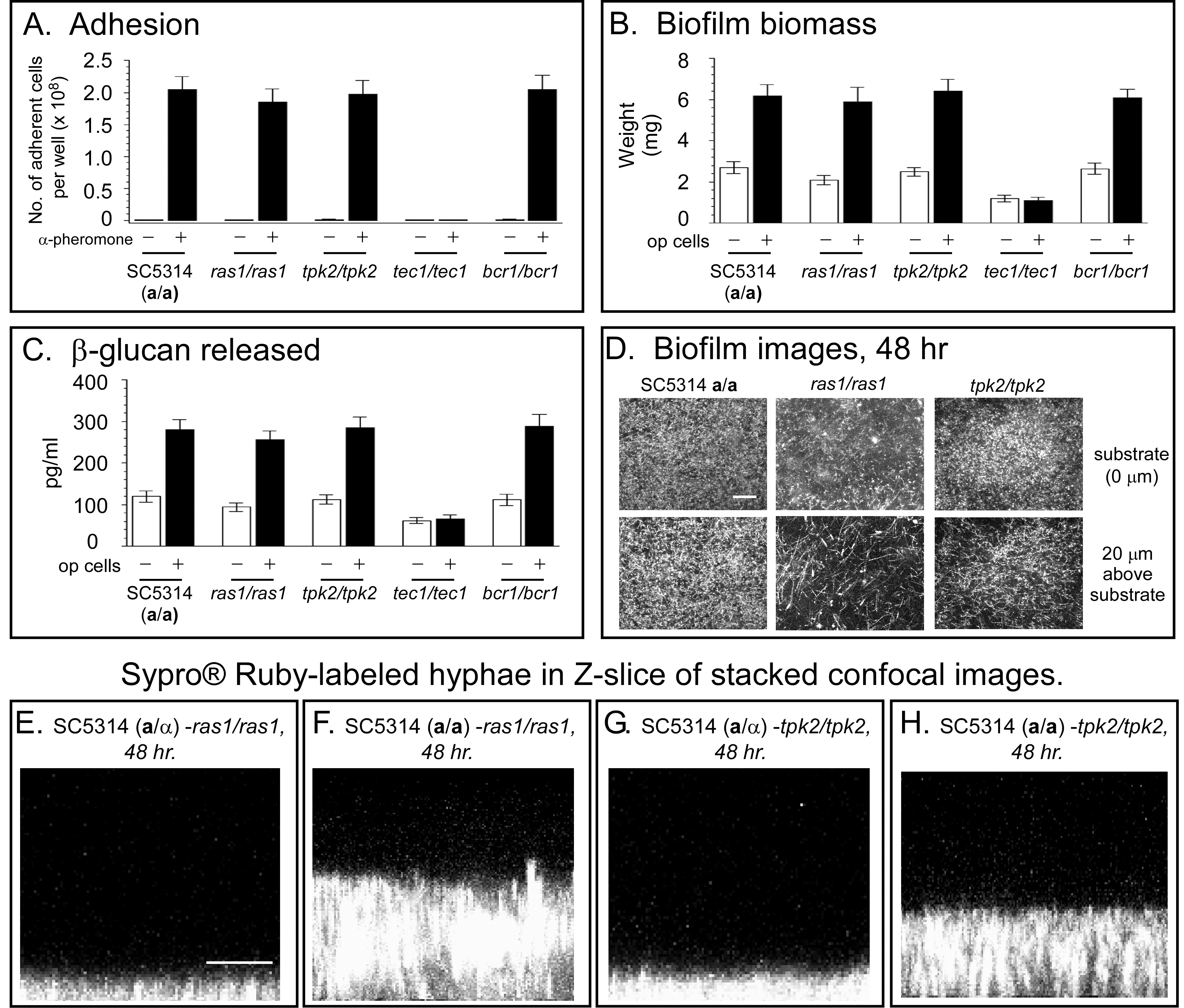

Supplement: Figure S1 — Ras1, Tpk2, and Bcr1 play no measurable role in a/a biofilm formation. Parental and mutant a/a strains are generated from a/a strains (see Table S2 for genotype origins and references). Methods can be found in Materials and Methods. Scale bar equals 100 µm. Note that in panels E and G, the use of a projection image obscures the true patchiness of the cell layers on the substratum. (TIF) [file pbio.1001117.s001.tif]

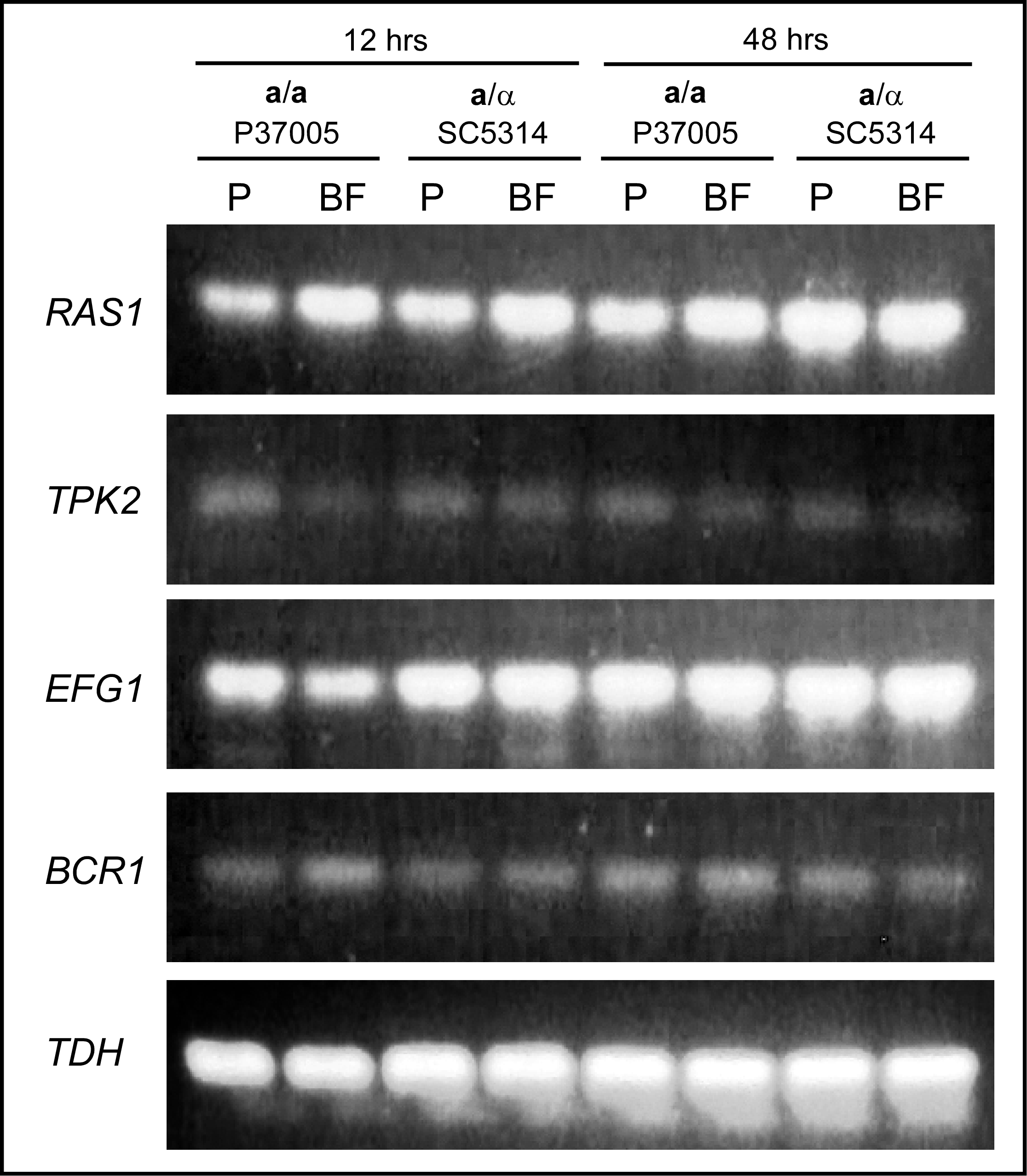

Supplement: Figure S2 — Expression of RAS1, TPK2, EFG1, and BCR1 in biofilms formed by a/a strain P37005 and a/a strain SC5314 under planktonic growth (P) and biofilm formation (BF) after 12 and 48 h of development. Methods can be found in Materials and Methods. TDH expression is known to be constitutive. (TIF) [file pbio.1001117.s002.tif]

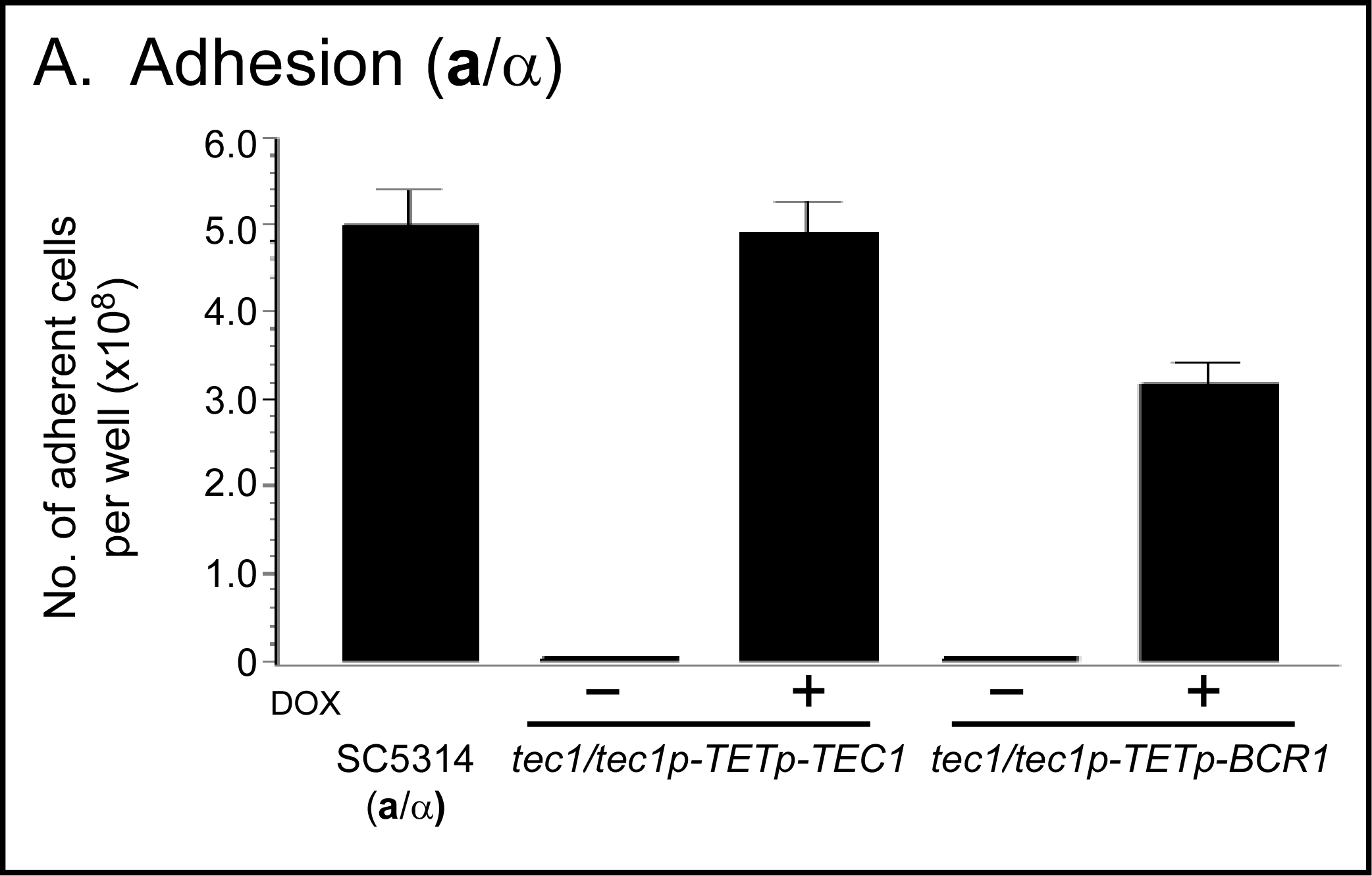

Supplement: Figure S3 — Overexpression of BCR1 in a tec1/tec1 mutant in a/a cells only partially rescues the defective adhesion phenotype. (TIF) [file pbio.1001117.s003.tif]

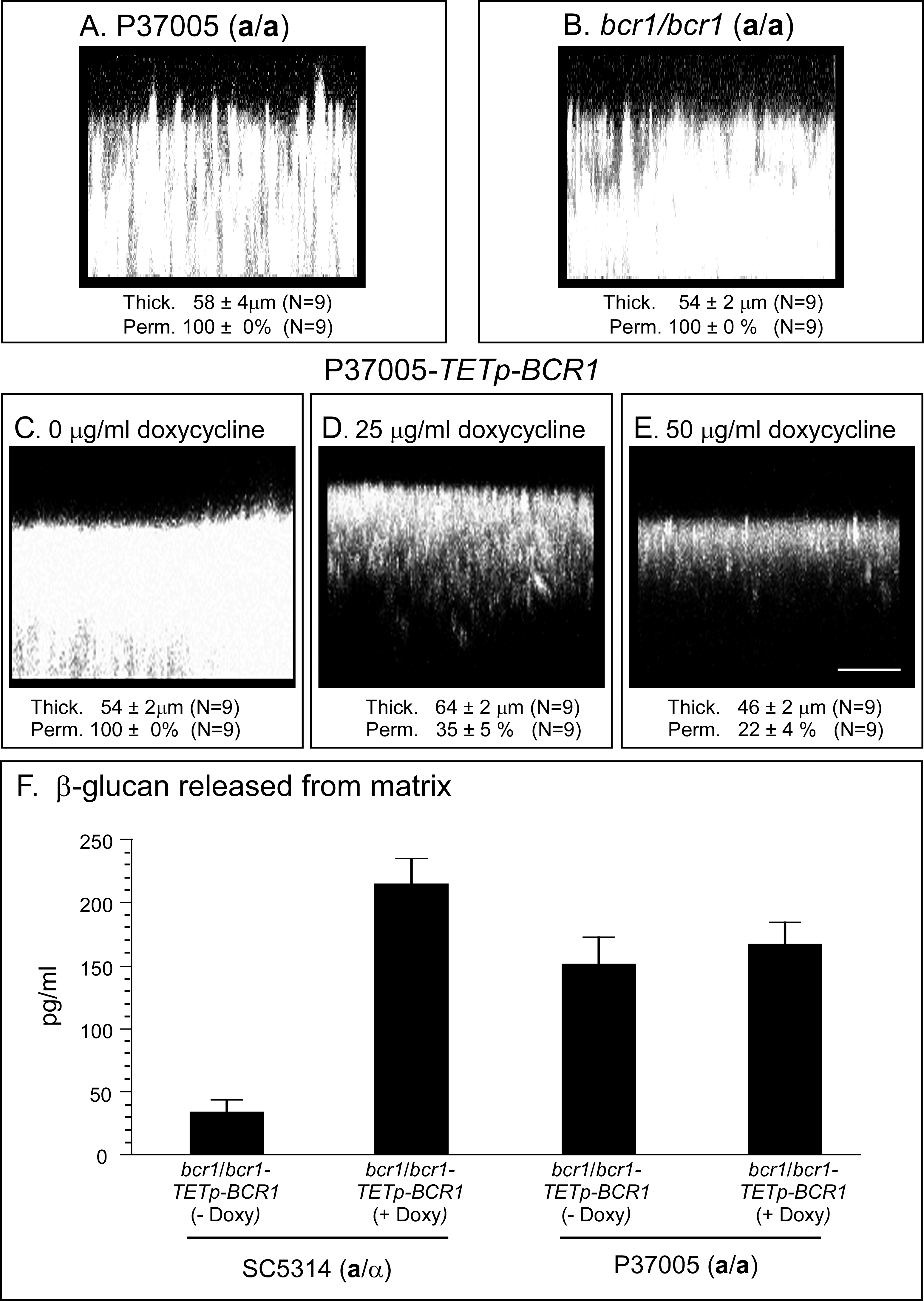

Supplement: Figure S4 — Overexpression of BCR1 in the a/a strain P37005 results in an increase in impermeability to Sypro Ruby. Thickness (Thick.) and permeability (Perm.) were quantitated. (A through E) Sypro Ruby staining of 48-h, live biofilms. (F) β-glucan released into medium. Scale bar equals 100 µm. (TIF) [file pbio.1001117.s004.tif]
