# Supplementary material for: Alternative Mating Type Configurations (a/α versus a/a or α/α) of Candida albicans Result in Alternative Biofilms Regulated by Different Pathways
Source: PLoS Biol. 2011 Aug 2;9(8):e1001117. doi: 10.1371/journal.pbio.1001117 (PMC3149048; doi:10.1371/journal.pbio.1001117)
Supplement: Table S1 — GFP-tagged strains used for biofilm dye permeability, cell penetration, and fluconazole susceptibility. (DOC) [file pbio.1001117.s005.doc]

**Supplemental Table S1. GFP-Tagged strains used for biofilm dye permeability, PMN penetration and fluconazole susceptibility.**

|  |  |  |  |  |
| --- | --- | --- | --- | --- |
| **Strain** | **Parent** | **MTL** | **Genotype** | **Source** |
| SC5314-GFP | SC5314 | **a**/α | *ADH1/adh1::pACT-GFP::SAT^R^* | This study |
| SC5314**a**-GFP | SC5314 | **a/a** | *ADH1/adh1::pACT-GFP::SAT^R^* | This study |
| SC5314α-GFP | SC5314 | α/α | *ADH1/adh1::pACT-GFP::SAT^R^* | This study |
| P37037-GFP | P37037 | **a**/α | *ADH1/adh1::pACT-GFP::SAT^R^* | [1] |
| P37037**a**-GFP | P37037 | **a/a** | *ADH1/adh1::pACT-GFP::SAT^R^* | This study |
| P37037α-GFP | P37037 | α/α | *ADH1/adh1::pACT-GFP::SAT^R^* | This study |
| P37039-GFP | P37039 | **a**/α | *ADH1/adh1::pACT-GFP::SAT^R^* | [1] |
| P37039**a**-GFP | P37039 | **a/a** | *ADH1/adh1::pACT-GFP::SAT^R^* | This study |
| P37039α-GFP | P37039 | α/α | *ADH1/adh1::pACT-GFP::SAT^R^* | This study |

1. Wu W, Lockhart SR, Pujol C, Srikantha T, Soll DR (2007) Heterozygosity of genes on the sex chromosome regulates *Candida albicans* virulence. Mol Microbiol 64: 1587-1604.

5.9.11
