# Supplementary material for: Alternative Mating Type Configurations (a/α versus a/a or α/α) of Candida albicans Result in Alternative Biofilms Regulated by Different Pathways
Source: PLoS Biol. 2011 Aug 2;9(8):e1001117. doi: 10.1371/journal.pbio.1001117 (PMC3149048; doi:10.1371/journal.pbio.1001117)
Supplement: Table S2 — Strains used in mutant studies. (DOCX) [file pbio.1001117.s006.docx]

**Supplemental Table S2. Strains used in mutant studies.**

|  |  |  |  |  |  |
| --- | --- | --- | --- | --- | --- |
| ***Strain*** | **Strain****name*** | **Parent** ***strain*** | **MTL** ***genotype*** | **Non-MTL genotype** | ***Source*** |
| *SC5314* | *─* | *─* | ***a****/α* | *Wild type* | *[2]* |
| *ste2/ste2* | *─* | *SC5314* | ***a****/α* | *ste2::FRT/ ste2::FRT* | *This study* |
| *ste11/ste11* | *─* | *SC5314* | ***a****/α* | *ste11::FRT/ ste11::FRT* | *This study* |
| *hst7/hst7* | *─* | *SC5314* | ***a****/α* | *hst7::FRT/ hst7::FRT* | *This study* |
| *cek1/cek1 cek2/cek2* | *─* | *SC5314* | ***a****/α* | *cek1::FRT/ cek1::FRT*  *cek2::FRT/ cek2::FRT* | *This study* |
| *cph1/cph1* | *JKC19* | *SC5314* | ***a****/α* | *ura3::λimm434/ura3::λimm434*  *cph1::hisG/cph1::hisG-URA3-hisG* | *[3]* |
| *SC5314-TETp-STE11* | *─* | *SC5314* | ***a****/α* | *ADH1/adh1::pTET-STE11-GFP::SAT^R^* | *This study* |
| *ras1/ras1* | *─* | *SC5314* | ***a****/α* | *ras1::FRT/ ras1::FRT* | *This study* |
| *ras1/ras1-TETp-RAS1* | *─* | *ras1/ras1* | ***a****/α* | *ras1::FRT/ ras1::FRT*  *ADH1/adh1::pTET-RAS1-GFP::SAT^R^* | *This study* |
| *ras1/ras1-TETp-RAS1V13* | *─* | *ras1/ras1* | ***a****/α* | *ras1::FRT/ ras1::FRT*  *ADH1/adh1::pTET-RAS1V13-GFP::SAT^R^* | *This study* |
| *cdc35/cdc35* | *CR216* | *SC5314* | ***a****/α* | *ura3::λimm434/ura3::λimm434 cdc35::hisG/cdc35:hisG-URA3-hisG* | *[4]* |
| *cdc35/cdc35-Metp-CDC35* | *─* | *cdc35/cdc35* | ***a****/α* | *ura3::λimm434/ura3::λimm434 cdc35::hisG/cdc35:hisG-URA3-hisG MET3/met3::pMet3-CDC35::SAT^R^* | *This study* |
| *pde2/pde2* | *─* | *SC5314* | ***a****/α* | *pde2::FRT/ pde2::FRT* | *This study* |
| *pde2/pde2-TETp-PDE2* | *─* | *pde2/pde2* | ***a****/α* | *pde2::FRT/ pde2::FRT*  *ADH1/adh1::pTET-PDE2-GFP::SAT^R^* | *This study* |
| tpk1/tpk1 | *─* | *SC5314* | ***a****/α* | *tpk1::FRT/ tpk1::FRT* | *This study* |
| *tpk1/tpk1-TETp-TPK1* | *─* | *tpk1/tpk1* | ***a****/α* | *tpk1::FRT/ tpk1::FRT*  *ADH1/adh1::pTET-TPK1-GFP::SAT^R^* | *This study* |
| tpk2/tpk2 | *─* | *SC5314* | ***a****/α* | *tpk2::FRT/ tpk2::FRT* | *This study* |
| *tpk2/tpk2-TETp-TPK2* | *─* | *tpk2/tpk2* | ***a****/α* | *tpk2::FRT/ tpk2::FRT*  *ADH1/adh1::pTET-TPK2-GFP::SAT^R^* | *This study* |
| efg1/efg1 | *MMY617* | SC5314 | **a**/α | *ura3::λimm434/ura3::λimm434 efg1::dpl200/efg1::URA3-dpl200* | *[5]* |
| *efg1/efg1-TETp-EFG1* | *─* | *efg1/efg1* | ***a****/α* | *ura3::λimm434/ura3::λimm434 efg1::dpl200/efg1::URA3-dpl200*  *ADH1/adh1::pTET-EFG1-GFP::SAT^R^* | *This study* |
| *ras1/ras1-TETp-EFG1* | *─* | *ras1/ras1* | ***a****/α* | *ras1::FRT/ ras1::FRT*  *ADH1/adh1::pTET-EFG1-GFP::SAT^R^* | *This study* |
| *cdc35/cdc35-TETp-EFG1* | *─* | *cdc35/cdc35* | ***a****/α* | *ura3::λimm434/ura3::λimm434 cdc35::hisG/cdc35:hisG-URA3-hisG ADH1/adh1::pTET-EFG1-GFP::SAT^R^* | *This study* |
| *tpk1/tpk1-TETp-EFG1* | *─* | *tpk1/tpk1* | ***a****/α* | *tpk1::FRT/ tpk1::FRT*  *ADH1/adh1::pTET-EFG1-GFP::SAT^R^* | *This study* |
| *tpk2/tpk2-TETp-EFG1* | *─* | *tpk2/tpk2* | ***a****/α* | *tpk2::FRT/ tpk2::FRT*  *ADH1/adh1::pTET-EFG1-GFP::SAT^R^* | *This study* |
| *tec1/tec1* | *─* | *SC5314* | ***a****/α* | tec1::FRT/ tec1::FRT | *This study* |
| *bcr1/bcr1* | *CJN702* | *SC5314* | ***a****/α* | *ura3::λimm434/ura3::λimm434*  *arg4::hisG his1::hisG bcr1::ARG4/* arg4::hisG his1::hisG bcr1::URA3 | *[6]* |
| *efg1/efg1-TETp-TEC1* | *─* | *efg1/efg1* | ***a****/α* | *ura3::λimm434/ura3::λimm434 efg1::dpl200/efg1::URA3-dpl200*  *ADH1/adh1::pTET-TEC1-GFP::SAT^R^* | *This study* |
| *efg1/efg1-TETp-BCR1* | *─* | *efg1/efg1* | ***a****/α* | *ura3::λimm434/ura3::λimm434 efg1::dpl200/efg1::URA3-dpl200*  *ADH1/adh1::pTET-BCR1-GFP::SAT^R^* | *This study* |
| *tec1/tec1-TETp-EFG1* | *─* | *tec1/tec1* | ***a****/α* | *tec1::FRT/ tec1::FRT*  *ADH1/adh1::pTET-EFG1-GFP::SAT^R^* | *This study* |
| *bcr1/bcr1-TETp-EFG1* | *─* | *bcr1/bcr1* | ***a****/α* | *ura3::λimm434/ura3::λimm434*  *arg4::hisG his1::hisG bcr1::ARG4/*  *arg4::hisG his1::hisG bcr1::URA3*  *ADH1/adh1::pTET-EFG1-GFP::SAT^R^* | *This study* |
| *efg1/efg1-TETp-EFG1T206A* | *─* | *efg1/efg1* | ***a****/α* | *ura3::λimm434/ura3::λimm434 efg1::dpl200/efg1::URA3-dpl200*  *ADH1/adh1::pTET-EFG1T206A-GFP::SAT^R^* | *This study* |
| *efg1/efg1-TETp-EFG1T206E* | *─* | *efg1/efg1* | ***a****/α* | *ura3::λimm434/ura3::λimm434 efg1::dpl200/efg1::URA3-dpl200*  *ADH1/adh1::pTET-EFG1T206E-GFP::SAT^R^* | *This study* |
| *P37005* | *─* | *─* | *a/a* | *Wild type* | *[7]* |
| *bcr1/bcr1* | *─* | *P37005* | *a/a* | *bcr1::FRT/ bcr1::FRT* | *This study* |
| *CAI4* | *─* | *SC5314* | ***a****/α* | *ura3::λimm434/ura3::λimm434* | *[8]* |
| *ras1/ras1* | *─* | *CAI4* | *a/a* | *ras1:hisG-URA3// ras1::hisG SOU+* | *This study* |
| *BWP17* | *─* | *CAI4* | ***a****/α* | *ura3D::imm434 his1::hisG arg4::hisG 27*  *ura3D::imm434 his1::hisG arg4::hisG* | *[9]* |
| *tpk2/tpk2* | *─* | *BWP17* | *a/a* | *tpk2::ARG4/ tpk2::HIS1 SOU+* | *[10]* |
| *P37005-TETp-BCR1* | *─* | *P37005* | *a/a* | *ADH1/adh1::pTET-BCR1-GFP::SAT^R^* | *This study* |
| *tec1/tec1*  *bcr1/bcr1*  *bcr1/bcr1-TETp-BCR1*  *bcr1/bcr1-TETp-BCR1* | —  —  —  — | *SC5314*  *SC5314*  *SC5314*  *P37005* | ***a/a***  ***a/a***  ***a/****α*  ***a/a*** | *tec1::FRT/tec1::FRT SOU+*  *bcr1::ARG4/bcr1::URA3 SOU+*  *bcr1::ARG4/bcr1::URA3*  *ADH1/adh1::pTET-BCR1-GFP::SAT^R^*  *bcr1:FRT/bcr1::FRT*  *ADH1/adh1::pTET-BCR1-GFP::SAT^R^* | *This study*  *This study*  *This study*  *This study* |
|  |  |  |  |  |  |
|  |  |  |  |  |  |

*Names from original reference, when noted

|  |  |
| --- | --- |

2. Gillum AM, Tsay EYH, Kirsch DR (1984). Isolation of the *Candida albicans* gene for orotidine-5’-phosphate decarboxylase by complementation of S. cerevisiae ura3 and E. coli pyrF mutations. Mol Gen Genet 198: 179-182.

3. Liu H, Köhler J, Fink GR (1994) Suppression of hyphal formation in *Candida albicans* by mutation of a STE12 homolog. Science 266: 1723-1726.

4. Cintia R. C. R, Schröppel K, Harcus D, Marcil A, Dignard D, et al. (2001) Signaling through adenylyl cyclase is essential for hyphal growth and virulence in the pathogenic fungus *Candida albicans*. Mol Biol Cell 12: 3631–3643.

5. Zordan RE, Miller MG, Galgozy DJ, Tuch BB, Johnson AD (2007) Interlocking transcriptional feedback loops control white-opaque switching in *Candida albicans*. PLoS Biol. 5: e256.

6. Nobile CJ, Mitchell AP (2005) Regulation of cell-surface genes and biofilm formation by the *C.albicans* transcription factor Bcr1p. Curr Biol 15: 1150-1155.

7. Lockhart R, Pujol C, Daniels KJ, Miller MG, Johnson AD, et al. (2002) In *Candida albicans*, white-opaque switchers are homozygous for mating type. Genetics 162: 737–745.

8. Fonzi WA, Irwin MY (1993) Isogenic strain construction and gene mapping in *Candida albicans.* Genetics 134: 717-728.

9. Wilson RB, Davis D, Mitchell AP. Rapid hypothesis testing with *Candida albicans* through gene disruption with short homology regions. Bacteriol. 181: 1868-1874.

10. Huang G, Yi S, Sahni N, Daniels KJ, Srikantha T, Soll DR (2010). N-Acetylglucosamine Induces white to opaque switching, a mating prerequisite in *Candida albicans*. PLoS Pathog*:* e1000806.

5.9.11
