# Supplementary material for: Alternative Mating Type Configurations (a/α versus a/a or α/α) of Candida albicans Result in Alternative Biofilms Regulated by Different Pathways
Source: PLoS Biol. 2011 Aug 2;9(8):e1001117. doi: 10.1371/journal.pbio.1001117 (PMC3149048; doi:10.1371/journal.pbio.1001117)
Supplement: Table S3 — Oligonucleotides used for mutant construction. (DOC) [file pbio.1001117.s007.doc]

|  | |  |
| --- | --- | --- |
|  |  |  |
| **Supplemental Table S3. Oligonucleotides used for mutant construction** | | |
|  |  |  |
| **Primer** | **Gene/Purpose** | **Sequence** |
| STE2f1 | *STE2* heterozygote | 5'-TCTATTGTGTAAACTATTAC-3' |
| STE2r1 | *STE2* heterozygote | 5-GTGTCCCGGGAATCAATGCCTAGTCGAT-3' |
| STE2f2 | *STE2* heterozygote | 5'-TGTACCCGGGCAAATCACCATCAAA-3' |
| STE2r2 | *STE2* heterozygote | 5'-CTTGTACTGGTTCAGCAACC-3' |
| STE2f3 | *STE2* homozygote | 5'-GATCGACTAGGCATTGATTTTTG-3' |
| STE2r3 | *STE2* homozygote | 5'-TCATCCCGGGTCTTCTTATGTTGAACAC-3' |
| STE2f4 | *STE2* homozygote | 5'-TCTTCCCGGGCTCAAACTGCTAATAAT-3' |
| STE2r4 | *STE2* homozygote | 5'-CACTCTTTTGATGGTGATTTG-3' |
| STE11f1 | *STE11* heterozygote | 5’-TGTTACTGCTCCACGTTATAA-3’ |
| STE11r1 | *STE11* heterozygote | 5’-TCCCCCGGGTCTCTGTCATGACTGTTTG-3’ |
| STE11f2 | *STE11* heterozygote | 5’-TCCCCCGGGTGAGTTGTCTTGTTTCTTTT-3’ |
| STE11r2 | *STE11* heterozygote | 5’-TCTTCGTATCCCGTTTCA-3’ |
| STE11f3 | *STE11* homozygote | 5’-TAACCCGAATAGATCGAAA-3’ |
| STE11r3 | *STE11* homozygote | 5’-TCCCCCGGGTAGGTACCAACATTATAC-3’ |
| STE11f4 | *STE11* homozygote | 5’-TCCCCCGGGAGTGGTTAAGCAAACTACA-3’ |
| STE11r4 | *STE11* homozygote | 5’-AATATCCAGAGAATGAGAAA-3’ |
| HST7f1 | *HST7* heterozygote | 5’-TTACTTACAAAGCCTTTGAT-3’ |
| HST7r1 | *HST7* heterozygote | 5’-TCCCCCGGGTACACCTTCTCCAATATCTC-3’ |
| HST7f2 | *HST7* heterozygote | 5’-TCCCCCGGGAACTTGTCACTGAATGAATA-3’ |
| HST7r2 | *HST7* heterozygote | 5’-TCCTGCTCAGAATAATGGCA-3’ |
| HST7f3 | *HST7* homozygote | 5’-ATATAAGTTGCGACGAAGCC-3’ |
| HST7r3 | *HST7* homozygote | 5’-TCCCCCGGGATTGTTGAGGTGGAGTATGA-3’ |
| HST7f4 | *HST7* homozygote | 5’-TCCCCCGGGTGGCAATTACTTGAAGACAA-3’ |
| HST7r4 | *HST7* homozygote | 5’-AGAGATGTTCAACTAGATT-3’ |
| CEK1f1 | *CEK1* heterozygote | 5'-ATTCCCGAGAATATATGA-3' |
| CEK1r1 | *CEK1* heterozygote | 5'-TCGCCCGGGTAAATAATATATAAGTTGA-3' |
| CEK1f2 | *CEK1* heterozygote | 5'-TCGCCCGGGTAAAGTTGAAGTTAAGTA-3' |
| CEK1r2 | *CEK1* heterozygote | 5'-GAGAGGTTTATTTGGTAGA-3' |
| CEK1f3 | *CEK1* homozygote | 5'-TTAAATTTACTATCCCAAA-3' |
| CEK1r3 | *CEK1* homozygote | 5'-TCGCCCGGGTGAGTTCTAATGACTCGAT-3' |
| CEK1f4 | *CEK1* homozygote | 5'-TCGCCCGGGTTGGTCAGTTGGTTGTAT-3' |
| CEK1r4 | *CEK1* homozygote | 5'-TCAAAACCTATACAACAA-3' |
| CEK2f1 | *CEK2* heterozygote | 5'-TAACGACAACTGCAGGAC-3' |
| CEK2r1 | *CEK2* heterozygote | 5'-TCACCCGGGTTGGTCAGGTATTGTAA-3' |
| CEK2f2 | *CEK2* heterozygote | 5'-TCACCCGGGCTTTACTTAATTAATTAC-3' |
| CEK2r2 | *CEK2* heterozygote | 5'-ACAATGGAGCACAATGCT-3' |
| CEK2f3 | *CEK2* homozygote | 5'-CTTCCTGTTACCATGTTA-3' |
| CEK2r3 | *CEK2* homozygote | 5'-TCACCCGGGCATGTATTCCTGAATAA-3' |
| CEK2f4 | *CEK2* homozygote | 5'-TCACCCGGGTTGAGTGCATCCAATTAT-3' |
| CEK2r4 | *CEK2* homozygote | 5'-CGACATGACTATTTCGA-3' |
| STE11f | *STE11* overexpression | 5'-TCCGTCGACAAAGATGACAGAGATTAATGATT-3' |
| STE11r | *STE11* overexpression | 5'-TCCGTCGACAATTGTTTCGACATAATTAATG-3' |
| RAS1f1 | *RAS1* heterozygote | 5’-TTATCTATGTATCATTGCCA-3’ |
| RAS1r1 | *RAS1* heterozygote | 5’-TCCCCCGGGGTTTAATTCTTGTCATGAG-3’ |
| RAS1f2 | *RAS1* heterozygote | 5’-TCCCCCGGGCCAAATGATCTTTCTGGTG-3’ |
| RAS1r2 | *RAS1* heterozygote | 5’-AACAATAACCCAAGTTACTT-3’ |
| RAS1f3 | *RAS1* homozygote | 5’-TTCTCAAACATTCTTCTGTTAATATA-3’ |
| RAS1r3 | *RAS1* homozygote | 5’-TCCCCCGGGATCAAGGTCAATGTCCAAT-3’ |
| RAS1f4 | *RAS1* homozygote | 5’-TCCCCCGGGCCAATTGATTGTTTCCAAGT-3’ |
| RAS1r4 | *RAS1* homozygote | 5’-AGAGGATGTTGAATGTAAGA-3’ |
| RAS1f | *RAS1* overexpression | 5'-TCCGTCGACAAAGATGTTGAGAGAATATAAATTAG-3' |
| RAS1r | *RAS1* overexpression | 5'-TCCGTCGACAAAACAATAACACAACATCCAT-3’ |
| RAS1V13f | *RAS1* hyperactivation | 5'-TTGTTGTTGGAGGT**GTT**GGTGTTGGTAAAT-3' |
| RAS1V13r | *RAS1* hyperactivation | 5'-ATTTACCAACACC**AAC**ACCTCCAACAACAA-3' |
| CDC35f | *CDC35* overexpression | 5’-ACAGATTGGATCCATGAGTTTTTTAAGGAGAG-3’ |
| CDC35r | *CDC35* overexpression | 5’-ACAGATTGCATGCTATCTATTTAAGTTCATTAA-3’ |
| PDE2f1 | *PDE2* heterozygote | 5’-ACAGACAACAACAACAACTA-3’ |
| PDE2r1 | *PDE2* heterozygote | 5’-TCCCCCGGGATATCTATTTTGTTGGTGTA-3’ |
| PDE2f2 | *PDE2* heterozygote | 5’-TCCCCCGGGTTGGATTATACAGATTAATA-3’ |
| PDE2r2 | *PDE2* heterozygote | 5’-TGTTGGATTACGATCTTTGT-3’ |
| PDE2f3 | *PDE2* homozygote | 5’-TTATCATTGGTTGACCTCGA-3’ |
| PDE2r3 | *PDE2* homozygote | 5’-TCCCCCGGGACAGCATAACACAACTGAGA-3’ |
| PDE2f4 | *PDE2* homozygote | 5’-TCCCCCGGGAAGAATATGTTGGCCAGATT-3’ |
| PDE2r4 | *PDE2* homozygote | 5’-CCAATTAAGTTTTATACTCTATAAG-3’ |
| PDE2f | *PDE2* overexpression | 5’-TCCGTCGACAAAGATGGCAGAAGTATTATCA-3’ |
| PDE2r | *PDE2* overexpression | 5’-TCCGTCGACAATTATTTCTTTGCTCTTTC-3’ |
| TPK1f1 | *TPK1* heterozygote | 5’-TTGGCGATTGTTTGAGTTTGT-3’ |
| TPK1r1 | *TPK1* heterozygote | 5’-TCCCCCGGGCAGATAATACTAGTCGGAAT-3’ |
| TPK1f2 | *TPK1* heterozygote | 5’-TCCCCCGGGTGTATTTATTGGCACAACAATGT-3’ |
| TPK1r2 | *TPK1* heterozygote | 5’-ATTCAAGAACGCGTTCGGTTAA-3’ |
| TPK1f3 | *TPK1* homozygote | 5’-AGATCAATATATCCTATCGTT-3’ |
| TPK1r3 | *TPK1* homozygote | 5’-TCCCCCGGGTTGCCTTTAGTTGTAGTGTC-3’ |
| TPK1f4 | *TPK1* homozygote | 5’-TCCCCCGGGATGTTAACTGGTTATACTCC-3’ |
| TPK1r4 | *TPK1* homozygote | 5’-AGTCCTGGAATTGATCACGA-3’ |
| TPK1f | *TPK1* overexpression | 5’-TCCGTCGACAAAGATGACATCCATGGAACCA-3’ |
| TPK1r | *TPK1* overexpression | 5’-TCCGTCGACAATTAAAAGTCCTGGAATTG-3’ |
| TPK2f1 | *TPK2* heterozygote | 5’-TGTTTAGTCTTAGATTTCCCAAC-3’ |
| TPK2r1 | *TPK2* heterozygote | 5’-TCCCCCGGGAGTGGGTGATTGTTTGTCTAA-3’ |
| TPK2f2 | *TPK2* heterozygote | 5’-TCCCCCGGGGGAGATGAACTTTCATAAATGA-3’ |
| TPK2r2 | *TPK2* heterozygote | 5’-AGCTATCACACTTACGACTCTT-3’ |
| TPK2f3 | *TPK2* homozygote | 5’-CACCTACTACTCACCCAATT-3’ |
| TPK2r3 | *TPK2* homozygote | 5’-TCCCCCGGGATCTACATGCTGCAATTGCT-3’ |
| TPK2f4 | *TPK2* homozygote | 5’-TCCCCCGGGAATGTTGGCAGGTTACAC-3’ |
| TPK2r4 | *TPK2* homozygote | 5’-AGTCAAGGAAATACAGAGCA-3’ |
| TPK2f | *TPK2* overexpression | 5’-TCCGTCGACAAAGATGGTGAATCTTTTAAAG-3’ |
| TPK2f | *TPK2* overexpression | 5’-TCCGTCGACAATCAAAAGTCAAGGAAATA-3’ |
| EFG1f | *EFG1* overexpression | 5'-TCCGTCGACAAAGATGTCAACGTATTCTATA-3' |
| EFG1r | *EFG1* overexpression | 5'-TCCGTCGACAAATGACTGAACTTGGGGTG-3' |
| TEC1f1 | *TEC1* heterozygote | 5'-TGTGTCTTGTGGTTAAGT-3' |
| TEC1r1 | *TEC1* heterozygote | 5'-TCCCCCGGGACAAATGTGAGATTGCAA-3' |
| TEC1f2 | *TEC1* heterozygote | 5'-TCCCCCGGGACTTACTCACTGTTGGAT-3' |
| TEC1r2 | *TEC1* heterozygote | 5'-TGATGCATTGAACAAGCT -3' |
| TEC1f3 | *TEC1* homozygote | 5'-ATGATGTCGCAAGCTACT -3' |
| TEC1r3 | *TEC1* homozygote | 5'-TCCCCCGGGTTCTGAATTTCCCGGTTT-3' |
| TEC1f4 | *TEC1* homozygote | 5'-TCCCCCGGGGAAAGTGAAGGTGGTCTTA-3' |
| TEC1r4 | *TEC1* homozygote | 5'-AAACTCACTAGTAAATCCT -3' |
| TEC1f | *TEC1* overexpression | 5'-TCCGTCGACAAAGATGATGTCGCAAGCTACT-3' |
| TEC1 | *TEC1* overexpression | 5'-TCCGTCGACAAAAACTCACTAGTAAATCC-3' |
| BCR1f1 | *BCR1* heterozygote | 5'-TTCAATTTCAGTTTACCTTGTT-3’ |
| BCR1r1 | *BCR1* heterozygote | 5'-TCCCCCGGGATTATTAATTGACTTGACAAACAA-3’ |
| BCR1f2 | *BCR1* heterozygote | 5’-TCCCCCGGGGATTGATTTTCAAGACTTGCA-3’ |
| BCR1r2 | *BCR1* heterozygote | 5’-CAGATCGGAATTTAACACT-3’ |
| BCR1f3 | *BCR1* homozygote | 5'-AGTAGCAGAATTCACTCAA-3’ |
| BCR1r3 | *BCR1* homozygote | 5'-TCCCCCGGGGATATATATCGAGATAGTATTAT-3’ |
| BCR1f4 | *BCR1* homozygote | 5'-TCCCCCGGGGAGAAGAAAGTATGTGAAAAATGTA-3’ |
| BCR1r4 | *BCR1* homozygote | 5’-TCATATCTTTACTCCTTTACTT-3’ |
| BCR1f | *BCR1* overexpression | 5'-TCCGTCGACAAAGATGTCAGGGACATCACAA-3' |
| BCR1r | *BCR1* overexpression | 5'-TCCGTCGACAATTGTGATATTAAATTATT-3' |
| Efg1T206Af | Efg1 phosphorylation | 5'-CAGACCACGAGTC**GCG**ACTACCATGTGG-3' |
| Efg1T206Af | Efg1 phosphorylation | 5'-CCACATGGTAGT**CGC**GACTCGTGGTCTG-3' |
| Efg1T206Ef | Efg1 phosphorylation | 5'-CAGACCACGAGTC**GAA**ACTACCATGTGG-3' |
| Efg1T206Er | Efg1 phosphorylation | 5'-CCACATGGTAGT**TTC**GACTCGTGGTCTG-3' |
| ACT1pf | *ACT1* promoter PCR | 5'-TCCGTCGACCCAGCCTCGTTTATAATAAAC-3’ |

| ACT1pr | *ACT1* promoter PCR | 5'-TCCGTCGACAATGATTATATTTTTTTAATATTAATATC-3’ |
| --- | --- | --- |
| MTLdel5'f | *MTL* locus deletion | 5’-CCCGTGGTAATACAAAAGAG-3' |
| MTLdel5'r | *MTL* locus deletion | 5’-ATTTCCCGGGAGGACAGAAGAACACAGA-3' |
| MTLdel3'f | *MTL* locus deletion | 5’-ATTTCCCGGGAAATCCAAAGGATCTTCTGCCACT-3' |
| MTLdel3'r | *MTL* locus deletion | 5’-TCAGAAATCCTGGGGTTAAGG-3' |
