# Supplementary material for: Alternative Mating Type Configurations (a/α versus a/a or α/α) of Candida albicans Result in Alternative Biofilms Regulated by Different Pathways
Source: PLoS Biol. 2011 Aug 2;9(8):e1001117. doi: 10.1371/journal.pbio.1001117 (PMC3149048; doi:10.1371/journal.pbio.1001117)
Supplement: Table S4 — Primer used for RT-PCR. (DOCX) [file pbio.1001117.s008.docx]

| **Supplemental Material** | | |
| --- | --- | --- |
| **Supplemental Table S4. Primers used for RT-PCR** | | |
|  |  |  |
| **Primer** | **Gene** | **Sequence** |
| BCR1-f | *BCR1* | 5'-TCACATGCAACAACATCAAC-3' |
| BCR1-r |  | 5'-AGAAGGTTGTTCTTGACCA-3' |
| SUN41-f | *SUN41* | 5’-GCTTGCCAAAGTGGTATG-3’ |
| SUN41-r |  | 5’-ACCTCTCCAAGTGTAATA-3’ |
| ALS3-f | *ALS3* | 5'-TATGACACCATGTCAAGT-3' |
| ALS3-r |  | 5'-AGCAGTAGTAAAAGTAGA-3' |
| ACT1 f | *ACT1* | 5'-TTGGTGTTTGACGAGTTT-3' |
| ACT1 r |  | 5'-TACCGTGTTCAATTGGGTAT-3' |
| BCR1 f2 | *BCR1* | 5’-AATGCVACTGCAGGTTATTTGG-3’ |
| BCR1 r2 |  | 5’-TTCTTGACCACCACCCATTT-3’ |
| EFG1 f | *EFG1* | 5’-TCAACAGGCTTTTCCTCAGC-3’ |
| EFG1 r |  | 5’-TGTTGACCTGGTTGTCCTTG-3’ |
| TDH3 f | *TDH3* | 5’-CGAAGGTGCTCAAAAACACA-3’ |
| TDH3 r |  | 5-TCAACGGTCTTTTGGGTAGC-3’ |
| RAS rtr | *RAS1* | 5-AAAAACCTTCACCGGTTCTC-3’ |
| RAS rtf |  | 5’-TGACCCAACTATTGAGGATTCTT-3’ |
| TPK2 rtf | *TPK2* | 5-AGAAACTTCACATCACCAAGCTG-3’ |
| TPK2 rtr |  | 5’-TGTTGTTGTTGATTGTCCA-3’ |

5.9.11
